# Adipocytes Encapsulating Telratolimod Recruit and Polarize Tumor‐Associated Macrophages for Cancer Immunotherapy

**DOI:** 10.1002/advs.202206001

**Published:** 2022-12-16

**Authors:** Di Wen, Tingxizi Liang, Guojun Chen, Hongjun Li, Zejun Wang, Jinqiang Wang, Ruxing Fu, Xiao Han, Tianyuan Ci, Yuqi Zhang, Peter Abdou, Ruoxin Li, Linlin Bu, Gianpietro Dotti, Zhen Gu

**Affiliations:** ^1^ Department of Bioengineering University of California Los Angeles California 90095 USA; ^2^ Earle A. Chiles Research Institute Robert W. Franz Cancer Center Providence Portland Medical Center Portland Oregon 97213 USA; ^3^ Department of Microbiology and Immunology School of Medicine University of North Carolina at Chapel Hill Chapel Hill NC 27599 USA; ^4^ Key Laboratory of Advanced Drug Delivery Systems of Zhejiang Province College of Pharmaceutical Sciences Zhejiang University Hangzhou 310058 China; ^5^ Jinhua Institute of Zhejiang University Jinhua 321299 P. R. China; ^6^ Department of General Surgery Sir Run Run Shaw Hospital School of Medicine Zhejiang University Hangzhou 310016 China

**Keywords:** adipocyte, cancer immunotherapy, drug delivery, macrophage

## Abstract

Tumor‐associated adipocytes (TAAs) recruit monocytes and promote their differentiation into tumor‐associated macrophages (TAMs) that support tumor development. Here, TAAs are engineered to promote the polarization of TAMs to the tumor suppressive M1 phenotype. Telratolimod, a toll‐like receptor 7/8 agonist, is loaded into the lipid droplets of adipocytes to be released at the tumor site upon tumor cell‐triggered lipolysis. Locally administered drug‐loaded adipocytes increased tumor suppressive M1 macrophages in both primary and distant tumors and suppressed tumor growth in a melanoma model. Furthermore, drug‐loaded adipocytes improved CD8^+^ T cell‐mediated immune responses within the tumor microenvironment and favored dendritic cell maturation in the tumor draining lymph nodes.

## Graphical abstract

The adipocytes are engineered to recruit macrophages and further deliver the lipid conjugated toll‐like receptor 7/8 agonist, which polarized the tumor‐associated macrophages to the M1 phenotype for cancer immunotherapy. By leveraging the tumor cell‐triggered lipolysis, a tumor‐responsive drug release is achieved and the systemic antitumor immune response suppressed both primary and distant tumor growth.

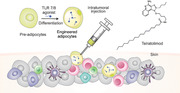

## 1 Introduction

Tumor‐associated adipocytes (TAAs) recruit circulating monocytes within the tumor and promote their differentiation into macrophages.^[^ ^1^, ^2^, ^3^, ^4^ ^]^ In turn, tumor‐associated macrophages (TAMs) exhibit a characteristic M2 phenotype, which promotes tumor development at least in part by inhibiting cytotoxic T lymphocytes (CTLs) and recruiting regulatory T cells (Tregs).^[^ ^5^, ^6^, ^7^ ^]^ In contrast, macrophages with M1 polarization secret pro‐inflammatory cytokines and contribute directly to eliminating tumor cells by phagocytosis.^[^ ^8^, ^9^ ^]^ In general, M2 macrophages prevail in many solid tumors, leading to an immunosuppressive tumor microenvironment (TME) that reduces the antitumor effects of immunotherapy.^[^ ^10^, ^11^ ^]^ Therefore, a variety of immunomodulatory agents have been developed to convert TAM from M2 polarization to the tumoricidal M1 phenotype. These agents modulate macrophages via binding with receptors such as toll‐like receptors (TLR), which recognize structurally conserved molecules and stimulate the innate immune response.^[^ ^12^, ^13^, ^14^ ^]^ Despite encouraging therapeutic outcome in ongoing clinical trials, the development of drug delivery approaches that specifically target and polarize TAMs within the tumor are desirable to enhance efficacy whilst controlling toxicity.^[^ ^15^ ^]^ Meanwhile, cytokine storm caused by TLR agonists needs to be carefully addressed to avoid severe morbidity and mortality.^[^ ^16^ ^]^

**Figure 1:**
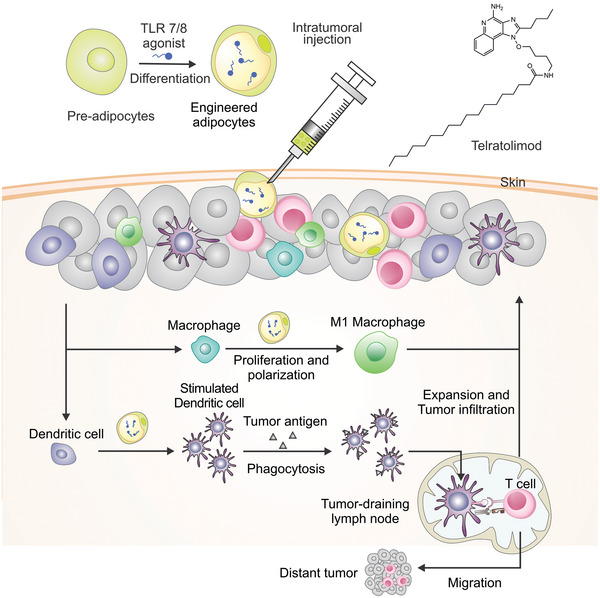
Schematic of the adipocyte‐based telratolimod drug delivery system for cancer immunotherapy. Engineered adipocytes recruit and stimulate the proliferation of macrophages that are also polarized to tumor suppressive M1 macrophages.

Herein, we described an adipocyte‐based drug delivery system that promotes the M1 polarization of TAMs (**Figure** **1**). Specifically, telratolimod, a lipid‐conjugated TLR 7/8 agonist, was encapsulated into the lipid droplets of adipocytes without compromising the lipid accumulation and physiologic functions of the adipocytes. Telratolimod‐loaded adipocytes boosted the recruitment of macrophages within the TME, released telratolimod via activation of the fatty acid binding protein 4, and induced M1 polarization of TAMs. Furthermore, telratolimod promoted the maturation of dendritic cells (DCs) within the tumor‐draining lymph nodes, leading to a subsequent T cell‐mediated antitumor immune response.

## 2 Results and Discussions

### 2.1 TLR 7/8 Agonist Induced the M1 Polarization of Macrophages

**Figure 2:**
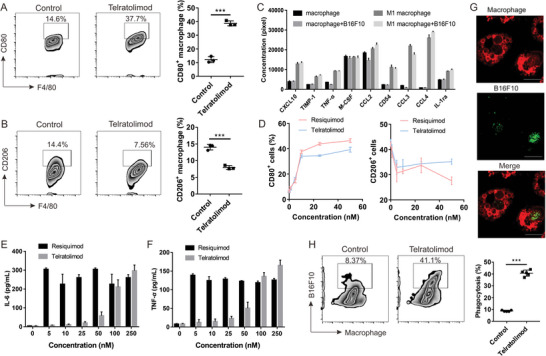
Telratolimod promoted M1 polarization and phagocytosis of macrophages. A,B) The subsets of M1 (A) and M2 (B) polarized macrophage were measured by flow cytometry. Bars represent means ± SD (*n* = 3). Student *t*‐test was performed. ***p < 0.001. C) Inflammatory cytokines were detected by cytokine array. Bars are presented as means ± SD (*n* = 3). D) M1 and M2 polarization effect of resiquimod and telratolimod. E,F) Inflammatory cytokines triggered by resiquimod and telratolimod were determined by ELISA. Bars are presented as means ± SD (*n* = 3). G,H) Phagocytosis of B16F10 cells by macrophages after treatment with telratolimod was determined by confocal microscopy (G) and flow cytometry (H). Bars represent means ± SD (*n* = 5). Student *t*‐test was performed. ***p < 0.001. Scale bar: 20 µm.

We co‐cultured bone marrow‐derived macrophages with B16F10 melanoma cells in a transwell system. B16F10 cells promoted the growth of macrophages (Figure S1A, Supporting Information) with M1 polarization (Figure S1B, Supporting Information) and decreased the number of macrophages with M2 phenotype (Figure S1C, Supporting Information). In contrast, macrophages suppressed melanoma cell growth without direct contact (Figure S1D, Supporting Information). TLR 7/8 is expressed in intracellular endosomes of macrophages, DCs, natural killer cells, and epithelial cells. Several small molecule TLR 7/8 agonists have been identified as potential anticancer agent via M1 macrophage polarization and increased phagocytosis.^[^ ^17^, ^18^ ^]^ Among these compounds, resiquimod has been applied to several clinical trials and the clinical efficacy has been demonstrated.^[^ ^19^, ^20^ ^]^ In this study, telratolimod, a small molecule analog of resiquimod with lipid conjugation, was used to polarize macrophages. This drug has been widely used as an adjuvant for the research of cancer immunotherapy and vaccines for HIV and SARS‐CoV‐2.^[^ ^21^, ^22^ ^]^ Similar to B16F10, telratolimod promoted the growth and M1 polarization of macrophages, while significantly decreased the subset of M2 macrophages (**Figure** **2**A,B; Figure S1A–D, Supporting Information). Activation of TLR 7/8 stimulates type 1 interferon‐mediated immune responses and anti‐tumor effects.^[^ ^23^, ^24^ ^]^ Therefore, we performed a cytokine array study and found that telratolimod induced inflammatory cytokine release (Figure 2C; Figure S1E, Supporting Information). These inflammatory cytokines secreted by macrophages, including CXCL10 and TNF‐ *α*, after co‐culturing with cancer cells may attributed to suppressed cancer cell growth as previously reported.^[^ ^25^, ^26^ ^]^ Meanwhile, CCL3 can enhance the function of CD8 T cells through IFN‐*γ* mediated DC recruitment.^[^ ^27^ ^]^ We then compared the capacity to promote M1 polarization of resiquimod and telratolimod. The macrophages exhibited similar M1 and M2 polarization after the treatment of both drugs (Figure 2D; Figure S2, Supporting Information), but resiquimod induced more pronounced secretion of inflammatory cytokines, including IL‐6 and TNF‐*α* (Figure 2E,F). The effect on phagocytosis capability of macrophages was also evaluated. Telratolimod significantly stimulated the phagocytosis of cancer cells by macrophages (Figure 2G,H; Figure S3, Supporting Information) without evidence of direct cytotoxicity toward B16F10 cells (Figure S4A, Supporting Information). Therefore, telratolimod exhibited anti‐cancer effects by inducing phagocytic property of macrophages and stimulating type 1 interferon‐mediated chemokine production.

### 2.2 Telratolimod‐Loaded Adipocytes Promoted M1 Polarization of Macrophages and Decreased Chemokine Production

**Figure 3:**
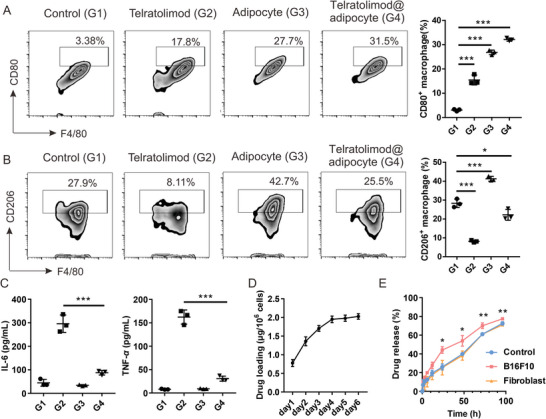
Telratolimod@adipocyte promoted M1 polarization while decreasing the cytokine release. A,B) M1 (A) and M2 (B) polarization after different treatment was measure by flow cytometry. C) Inflammatory cytokines were determined by ELISA. Data are presented as means ± SD (*n* = 3). One‐way ANOVA with a Tukey post‐hoc test was performed. *p < 0.05, ***p < 0.001. G1: Control. G2: Telratolimod (100 nM). G3: Adipocytes. G4: Telratolimod@adipocyte. D,E) Loading capacity (D) and release profile (**E**) of telratolimod by the adipocytes were determined by HPLC. “Control” represents telratolimod@adipocyte alone in the transwell. Data are presented as means ± SD (*n* = 3). One‐way ANOVA with a Tukey post‐hoc test was performed to compare the statistical significance between control and B16F10 cell co‐cultured group at different time points. *p < 0.05, **p < 0.01.

To further improve the therapeutic index of telratolimod, we explored the use of adipocytes as drug delivery vehicles ^[^ ^28^ ^]^ to recruit and polarize macrophages within the TME.^[^ ^1^, ^29^, ^30^ ^]^ We hypothesize that higher anti‐cancer efficacy could be achieved by using adipocytes as drug delivery vehicle due to i) the high loading capacity and biocompatibility of adipocytes for the delivery of telratolimod; ii) bioresponsive drug release profile induced by tumor triggered lipolysis; iii) synergistic effect of engineered adipocytes in boosting TAMs. When normally differentiated adipocytes were co‐cultured with macrophages in a transwell assay, we observed significant increase in both M1 and M2 polarized macrophages (**Figure** **3**A,B). Telratolimod‐loaded adipocytes exhibited enhanced capacity to promote M1 polarization, but showed negligible effects on M2 polarization. Since telratolimod alone caused a significant reduction of M2 polarization of macrophages, telratolimod‐loaded adipocytes may at least in part block the intrinsic property of adipocytes to promote M2 polarization. The encapsulation of telratolimod did not alter the adipokine profile of adipocytes (Figure S5, Supporting Information), suggesting that this small molecule does not interfere with their growth or function. However, adipocytes significantly inhibited the secretion of cytokines from macrophages in the presence of telratolimod (Figure 3C). In accordance with our previous study,^[^ ^28^, ^31^ ^]^ lipid modification significantly enhanced the drug loading capacity of telratolimod within the lipid droplets (Figure 3D), whereas resiquimod exhibited nondetectable loading amount in adipocytes (Figure S4B,C, Supporting Information). Moreover, B16F10 caused the release of the drug from adipocytes compared with fibroblasts (Figure 3E), indicating the tumor‐responsive manner of the adipocyte‐based drug delivery system. Furthermore, the fatty acid binding protein 4 (FABP4) inhibitor reversed the M1 polarization of macrophage when co‐cultured with telratolimod@adipocytes in transwell (Figure S6, Supporting Information), indicating that the delivery of telratolimod from adipocytes is mediated by FABP4.

Cancer cells can generate a suppressive TME by recruiting several types of nonmalignant cells that promote angiogenesis and suppress anti‐cancer immune responses.^[^ ^32^, ^33^, ^34^ ^]^ We sought to elucidate the interaction among cancer cells, adipocytes, macrophages, and effector T cells in response to telratolimod. Similar to the adipocytes, CD3^+^ cells promoted both M1 and M2 polarization of macrophages (Figure S7A,B, Supporting Information, group 2). However, B16F10 cells significantly inhibited the M1 polarization (Figure S7A, Supporting Information, group 2 and group 5) and promoted the M2 polarization of macrophages (Figure S7B, Supporting Information, group 6 and group 8) when adipocytes were present. Telratolimod decreased the M2 polarization of macrophage when we compare the population of CD206 positive macrophage in each group between Figures S7B,D. Specifically, the M2 polarization of macrophages when co‐cultured with cancer cells, CD3^+^ T cells, and adipocytes was significantly inhibited by telratolimod (Figures S7B,S7D, group 7 and group 8). With decreased M2 polarization, the major macrophages were switched to the M1 phenotype. We further determined if Tregs may be involved. Adipocytes and cancer cells could work together to increase Treg (Figure S8A, Supporting Information, group 2, group 4, and group 5). Interestingly, adipocytes and macrophages enhanced the Treg population to balance the inflammatory reaction when telratolimod was presented (Figure S8B, Supporting Information, group 2, group 3, and group 7). In contrast, this process was significantly reversed when B16F10 cells were added, suggesting that telratolimod can effectively decrease Tregs in TME (Figure S8B, Supporting Information, group 7 and group 8). We also measured the cytokine levels in the medium and found that adipocytes exhibited the strongest anti‐inflammatory effect compared with cancer cells and T cells (Figures S7E,F and S8C,D, Supporting Information). Therefore, telratolimod delivery by adipocytes induced a specific remodeling of TME with an enhanced ratio of M1/M2 macrophage, which can be translated in the local and distal control of tumor growth.

### 2.3 Telratolimod@adipocyte Induced an Immunogenic Tumor Phenotype and Suppressed Tumor Growth

**Figure 4:**
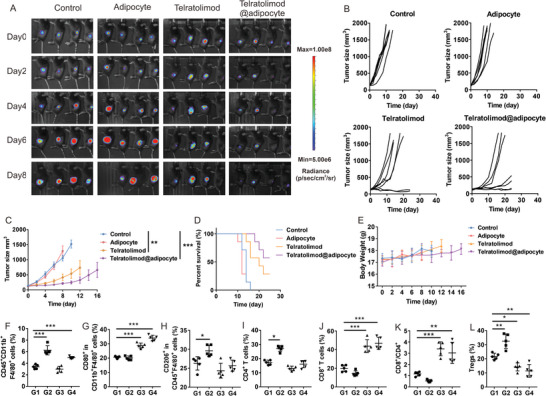
Telratolimod@adipocyte suppressed local tumor growth. A) Representative tumor bioluminescence in each group. B) Individual tumor growth. C) Average tumor size in each experimental group. Data are presented as means ± SEM (*n* = 6). Statistical significance was calculated via one‐way ANOVA analysis with a Tukey post‐hoc test. **p < 0.01, ***p < 0.001. D) Survival curves of the mice in each group. **E**) Average body weight of mice in each experimental group. F–L) Quantity of immune cells including TAMs (F), M1 macrophages (G), M2 macrophages (H), CD4^+^ T cells (I), CD8^+^ T cells (J), CD8/CD4 ratio (K), and Tregs (L) was determined by flow cytometry. G1: Control. G2: Adipocytes. G3: Telratolimod (0.2 mg kg^−1^). G4: Telratolimod@adipocyte. Data are presented as means ± SD (*n* = 5). Statistical significance was calculated via one‐way ANOVA analysis with a Tukey post‐hoc test. *p < 0.05, **p < 0.01, ***p < 0.001.

We further validated the therapeutic effect of Telratolimod@adipocytes in vivo using the B16F10 mouse melanoma model. We first evaluated the pharmacokinetics profile of telratolimod after in vivo administration (Figure S9, Supporting Information). The profiles of telratolimod concentration in the serum were similar after subcutaneous injection of free drug in normal mice and tumor‐bearing mice with the highest concentration reached within 12 h. In contrast, telratolimod@adipocytes exhibited a controlled‐release profile. Notably, we observed significantly promoted drug release after intratumoral injection of telratolimod@adipocytes in tumor‐bearing mice compared with normal mice. Telratolimod exhibited enhanced antitumor effects when delivered as telratolimod@adipocytes compared with free drug (**Figure** **4**A–C), leading to improved survival of the mice (Figure 4D) in the absence of systemic toxicity (Figure 4E). In separated experiments, tumors were collected and analyzed by flow cytometry and immunofluorescence staining. Adipocyte or telratolimod@adipocyte caused significant increase of TAM population in the TME (Figure 4F; Figure S10A, Supporting Information). In particular, M1 macrophages were increased after treatment with free telratolimod or telratolimod@adipocytes (Figure 4G). In contrast, in tumors treated with telratolimod we did not observe modifications of M2 macrophage population, which was increased in tumors treated with adipocytes alone (Figure 4H). CD4^+^ T cells were also increased after the treatment with adipocytes (Figure 4I; Figure S10B, Supporting Information), whereas telratolimod enhanced the infiltration of CD8^+^ T cells (Figure 4J; Figure S10B, Supporting Information) and increased the CD8^+^/CD4^+^ ratio (Figure 4K). In accordance with the in vitro results (Figure S8A, Supporting Information), adipocytes enhanced the Treg recruitment within the TME, while Treg content was decreased after the treatment of telratolimod (Figure 4L). The administration of adipocytes decreased the inflammatory cytokine levels in the plasma, which was reversed by local administration of telratolimod (Figure S11, Supporting Information).

**Figure 5:**
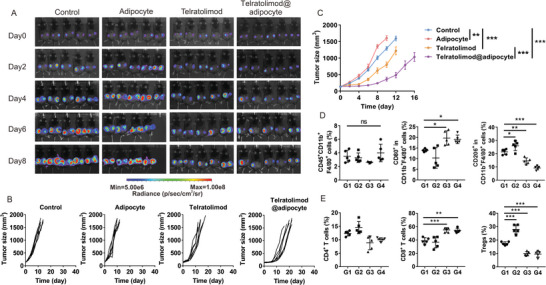
Telratolimod@adipocyte suppressed tumor growth at distant sites. A) Representative figures of tumor bioluminescence in each group. B) Individual distant tumor growth. C) Average distant tumor size in each group. D,E) Quantification of macrophages (D) and T cells (E) in the distant tumor. G1: Control. G2: Adipocytes. G3: Telratolimod (0.2 mg kg^−1^). G4: Telratolimod@adipocyte. Data are presented as means ± SD (*n* = 5). Statistical significance was calculated via one‐way ANOVA analysis with a Tukey post‐hoc test. *p < 0.05, **p < 0.01, ***p < 0.001.

After showing that telratolimod induced a strong local immune response, we investigated whether local treatment could cause antitumor effects in distant tumor lesions. In mice inoculated with B16F10 cells in both flanks, different treatment modalities were only applied on the right flank. Both original and distant tumor growth was significantly inhibited when free telratolimod or telratolimod@adipocytes were administrated (**Figure** **5**A–C; Figure S12A,B, Supporting Information) without significant modification of body weight as measurement of systemic toxicity (Figure S12C, Supporting Information). TAMs were significantly increased within the primary tumor treated with adipocytes or telratolimod@adipocytes (Figure S12D, Supporting Information). Although adipocyte administration did not significantly increase TAMs in the distant tumors, the percentage of M1 macrophages was significantly enhanced (Figure 5D). This systemic immune response was further confirmed by the increase of CD8^+^ T cells in both original and distant tumors associated with decreased Tregs population (Figure 5E; Figure S12E, Supporting Information). Administration of adipocytes or telratolimod did not result in evidence of side effects to major organs (Figure S13, Supporting Information). The previous report indicated that TLR 7/8 agonist including resiquimod promoted DC maturation and thereby augment immune response against tumor growth.^[^ ^35^, ^36^ ^]^ To further demonstrate the induction of a systemic immune response, we isolated the tumor‐draining lymph nodes of both primary and distant tumors and observed the presence of activated DCs, which suggest the occurrence of antigen‐cross presentation (Figure S14, Supporting Information). Since TLR 7/8 is also expressed within the endosome of DCs^[^ ^35^, ^37^ ^]^ and telratolimod significantly promoted the expression of CD80/CD86 in DCs isolated from both primary and distant tumor‐draining lymph nodes (Figure S14, Supporting Information), DC cross‐priming mediated by telratolimod@adipocytes may account for the systemic immunologic effects observed upon local tumor treatment. Of note, we also observed decreased IL‐6 and IL‐10 as well as increased TNF‐*α* after the treatment with telratolimod, which might contribute to the inhibition of distant tumor growth (Figure S15, Supporting Information). Previous studies indicated that IL‐12 secreted by macrophages attributed to the anti‐cancer effect of M1 macrophage.^[^ ^38^ ^]^ However, telratolimod did not promote the secretion of IL‐12 in vitro and in vivo, whereas we did not observe significant difference of IL‐12 levels in both primary and distant tumor models (Figures S11 and S15, Supporting Information).

To further confirm the systemic immune response after the administration of telratolimod or telratolimod@adipocytes, we evaluated their therapeutic efficacy in a distant 4T1 mammary carcinoma model. Both original and distant tumor growth was significantly inhibited after the administration of free telratolimod or telratolimod@adipocytes (Figures S16A,B and Figure S17A,B, Supporting Information). Similar to the distant mouse melanoma model, telratolimod and telratolimod@adipocytes enhanced the population of M1 macrophages and effector T cells in primary tumor and decreased M2 macrophages (Figure S16C, Supporting Information). The systemic immune response was confirmed by the similar immune cell population in distant tumor (Figure S17C, Supporting Information). Other than macrophage polarization, telratolimod and telratolimod@adipocytes promoted the maturation of DC in both tumors. (Figures S16C and S17C, Supporting Information) The systemic toxicity was further evaluated by complete blood count (Figure S18, Supporting Information), showing no detectable side effects after the administration of telratolimod or engineered adipocytes whereas the population of white blood cells was above the normal range after tumor inoculation.

## 3 Conclusion

In this study, we reported a new strategy for cell‐based immunotherapy by leveraging the ability of adipocytes to recruit macrophages and promote their M1 polarization within the TME using a TLR 7/8 agonist. The proposed strategy showed a broad remodeling of the TME that also included decreased population of Tregs and activation of DCs. Due to the fat embolism syndrome caused by the abnormal infiltration of lipid droplets in blood vessel, engineered adipocytes are more suitable for intratumoral or peri‐tumoral rather than systemic injection. Moreover, most of the clinical tests of TLR 7/8 agonist are through local injection, probably because the TLR 7/8 is expressed by a variety of cell types and the severe systemic immune response needs to be avoided. In this research, the local delivery of telratolimod provided by telratolimod@adipocytes may attenuate the cytokine storm caused by systemic delivery of TLR agonist even if the long‐term safety of local adipocyte administration needs to be thoroughly evaluated in controlled clinical studies. Taking into consideration the loading capacity of adipocytes, multiple immunomodulatory agents could be accommodated to further increase the potency of immune response. Furthermore, isolation and engineering of adipocytes are relatively simple and robust approaches, which can be easily translated in clinical applications.

## 4 Experimental Section

### Materials and Cell Lines

All chemicals were purchased from Sigma–Aldrich and used as received unless specified. Telratolimod (MEDI9197) was purchased from MedChemExpress. 3T3‐L1 (CL‐173) and B16F10 (CRL‐6475) were purchased from the American Type Culture Collection. Bioluminescent B16F10 cells (B16F10‐luc‐GFP) were provided by Dr. Leaf Huang from the University of North Carolina at Chapel Hill. B16F10 was cultured in RPMI 1640 (Gibco, Invitrogen) with 10% FBS (Invitrogen). Mouse primary dermal fibroblast was purchased from Cell Biologics (catalog no. C57‐6067) and cultured using Fibroblast Medium Kit (catalog no. M2267). Mouse primary macrophage was isolated from the bone marrow of C57BL/6J mouse and cultured in RPMI 1640 with 10%FBS and M‐CSF (20 ng mL^−1^) for 4 days before experiments. 3T3‐L1 was cultured in DMEM with 10% bovine calf serum (Thermo Fisher Scientific). For differentiation, a 3T3‐L1 Differentiation Kit (Sigma–Aldrich catalog no. DIF001) was used to achieve the maximum accumulation of lipid droplets.

### Loading and Release of Resiquimod and Telratolimod

For generating resiquimod or telratolimod‐loaded adipocytes, resiquimod or telratolimod (5 µM) were added in the maintenance medium (DMEM/F12 (1:1) with 10% FBS and 1.5 mg mL^−1^ insulin) and changed for every 48 h. Preadipocytes were cultured, differentiated, and drug encapsulated in 6‐well transwell insert, and co‐cultured with 5 × 10^5^ precultured B16F10 or fibroblasts in 6 well plate to determine drug release profiles, which was calculated according to drug amount remained in adipocytes. To measure the amount of telratolimod in adipocytes, 20 µL Triton X‐100 (10%) was added to 10^6^ adipocytes. Then, 100 µL extraction solution (0.75 M HCl in isopropanol) was added and incubated at −20 °C overnight. The concentration of telratolimod was measured by HPLC. The maintenance medium was changed three to four times for animal work. To determine the stability of telratolimod in adipocytes, the amount of telratolimod was measured at 0 and 24 h in telratolimod@adipocytes by HPLC. Since the concentration of released telratolimod in cell culture medium is not detectable, it was measured the remained drug amount in telratolimod@adipocytes to determine the loading and release profiles of telratolimod. HPLC method for telratolimod: Agilent C18 column 4.6 × 50 mm eluted with water and acetonitrile (starting 95:5, and then after 6‐min gradient up to 5:95).

### Crosstalk Between Cancer Cell and Adipocyte

Cytotoxicity of drug was determined by MTT assay in 96‐well plate after 48 h. Crosstalk among adipocyte, B16F10 (1 × 10^5^/well), and immune cells was determined in a transwell system where macrophages (2 × 10^4^/well) and T cells (4 × 10^4^/well) were seeded in the 6 well plate while tumor cells and adipocytes or telratolimod@adipocytes (1 × 10^5^/well) grew in the transwell insert.^[^ ^39^, ^40^, ^41^ ^]^ CD4 and CD8 cells were isolated from C57BL/6 mouse spleen using EasySep Mouse CD4^+^/CD8^+^ T Cell Isolation Kit (Stemcell Technologies) and cultured in RPMI1640 medium with 10% FBS. After culturing for 72 h, the polarization of macrophages and the population of Treg were determined by flow cytometry. The concentration of IL‐6, IL‐10, and TNF‐*α* was determined by ELISA.

For adipokine profiling (R&D Systems catalog no. ARY013), 6 well transwell system was used with cancer cells cultured in the transwell insert and adipocytes in the bottom. Cells or medium were analyzed after co‐culturing for 72 h. To determine the role of FABP4 during the crosstalk, 30 µM BMS309403 was added in the medium to block FABP4.

### In Vivo Tumor Studies

All animal studies were carried out following protocols approved by the Institutional Animal Care and Use Committee at the University of California, Los Angeles, and Earle A. Chiles Research Institute. Female C57BL/6 mice (6–8 weeks) and BALB/c mice (6–8 weeks) were purchased from the Jackson Lab. For a subcutaneous tumor models, 1 × 10^6^ luciferase‐tagged B16F10 cells (melanoma model) or 1 × 10^6^ 4T1 cells (mammary carcinoma model) were injected into the right flank of mice. When the tumor reached 50–100 mm^3^, mice were randomly divided into four groups (*n* = 10–11) with intratumorally injected different formulations on day 0 and day 3, including PBS, normally differentiated adipocytes, free telratolimod, and telratolimod@adipocytes. The doses of telratolimod was 0.2 mg kg^−1^ (usually 5 × 10^6^ adipocytes) for anticancer analysis and 0.4 mg kg^−1^ (usually 1 × 10^7^ adipocytes) for pharmacokinetics studies. Tumor size was measured with a digital caliper and monitored by bioluminescence signal using IVIS Lumina imaging system (PerkinElmer) with intraperitoneal injection of luciferin (catalog no. LUCK‐100, Gold Biotechnology) at 150 mg kg^−1^. Tumor volume was calculated as long diameter × short diameter^2^/2.

For a distant tumor model,^[^ ^42^ ^]^ 1 × 10^6^ luciferase expressing B16F10 cells (melanoma model) or 1 × 10^6^ 4T1 cells (mammary carcinoma model) were subcutaneously inoculated into both left and right flanks of mice. After randomly dividing the mice into different groups (*n* = 10–12), tumors in the right flank were treated with PBS, normally differentiated adipocytes, free telratolimod, and telratolimod@adipocytes. Tumor growth was monitored by detecting the bioluminescence and measuring tumor size. For both intratumoral and distant tumor models, mice were euthanized when the tumor size exceeded 1.5 cm^3^.

To determine the population of immune cells in tumor, 4–5 mice were sacrificed in each group to obtain the tumors two days after the second injection of the formulation. The same time point was utilized to determine the cytokine levels in blood serum. A single‐cell suspension of tumor was prepared using staining buffer (catalog 420 201, BioLegend). 20 000 events per sample were collected and analyzed using FlowJo software. Antibodies for detecting CD4^+^ T cells, CD8^+^ T cells, and Tregs included CD3 (catalog 100 203, Biolegend), CD4 (catalog 100 515, Biolegend), CD8 (catalog 100 707, Biolegend), FoxP3 (catalog 126 403, Biolegend).

### Statistics

One‐way ANOVA and a Tukey post‐hoc test were performed for multiple comparisons. Bars represent means ± SD or means ± SEM as noted in the figure legends. All tests were performed using the GraphPad Prism software. Significance was defined as *p < 0.05, **p < 0.01, ***p < 0.001.

## Conflict of Interest

Z.G. and D.W. have applied for patents related to this study. Z.G. is a scientific co‐founder of ZenCapsule Inc., ZCapsule Inc., Zenomics Inc., and Wskin Inc.

## Author Contributions

D.W. and Z.G. were responsible for the conception and experimental strategy of the study. D.W., L.T., G.C., H.L., Z.W., R.F., X.H., T.C., R.L., and P.A. performed the experiments and acquired the data. D.W., T.L., G.C., H.L., Z.W., J.W., T.C., R.L., P.A., and Z.G. interpreted the data and drafted the manuscript. G.C., H.L., Z.W., Y.Z., P.A., L.B., G.D., and Z.G. supported the revision of the manuscript.

## Supporting information

Supporting Information

## Data Availability

The data that support the findings of this study are available from the corresponding author upon reasonable request.

## References

[1] L. H. Correa , R. Correa , C. M. Farinasso , L. P. de Sant'Ana Dourado , K. G. Magalhaes , Front. Immunol. 2017, 8, 1129. 28970834 10.3389/fimmu.2017.01129 PMC5609576

[2] D. G. DeNardo , B. Ruffell , Nat. Rev. Immunol. 2019, 19, 369. 30718830 10.1038/s41577-019-0127-6 PMC7339861

[3] C. E. Lewis , J. W. Pollard , Cancer Res. 2006, 66, 605. 16423985 10.1158/0008-5472.CAN-05-4005

[4] J. W. Pollard , Nat. Rev. Cancer 2004, 4, 71. 14708027 10.1038/nrc1256

[5] T. J. Curiel , G. Coukos , L. Zou , X. Alvarez , P. Cheng , P. Mottram , M. Evdemon‐Hogan , J. R. Conejo‐Garcia , L. Zhang , M. Burow , Y. Zhu , S. Wei , I. Kryczek , B. Daniel , A. Gordon , L. Myers , A. Lackner , M. L. Disis , K. L. Knutson , L. Chen , W. Zou , Nat. Med. 2004, 10, 942. 15322536 10.1038/nm1093

[6] M. Ovais , M. Guo , C. Chen , Adv. Mater. 2019, 31, 1808303. 10.1002/adma.201808303 30883982

[7] M. De Palma , D. Biziato , T. V. Petrova , Nat. Rev. Cancer 2017, 17, 457. 28706266 10.1038/nrc.2017.51

[8] J. M. Brown , L. Recht , S. Strober , Clin. Cancer Res. 2017, 23, 3241. 28341752 10.1158/1078-0432.CCR-16-3122 PMC5529121

[9] A. Mantovani , F. Marchesi , A. Malesci , L. Laghi , P. Allavena , Nat. Rev. Clin. Oncol. 2017, 14, 399. 28117416 10.1038/nrclinonc.2016.217 PMC5480600

[10] S. P. Arlauckas , C. S. Garris , R. H. Kohler , M. Kitaoka , M. F. Cuccarese , K. S. Yang , M. A. Miller , J. C. Carlson , G. J. Freeman , R. M. Anthony , R. Weissleder , M. J. Pittet , Sci. Transl. Med. 2017, 9, eaal3604. 28490665 10.1126/scitranslmed.aal3604 PMC5734617

[11] S. R. Gordon , R. L. Maute , B. W. Dulken , G. Hutter , B. M. George , M. N. McCracken , R. Gupta , J. M. Tsai , R. Sinha , D. Corey , A. M. Ring , A. J. Connolly , I. L. Weissman , Nature 2017, 545, 495. 28514441 10.1038/nature22396 PMC5931375

[12] C. B. Rodell , S. P. Arlauckas , M. F. Cuccarese , C. S. Garris , R. Li , M. S. Ahmed , R. H. Kohler , M. J. Pittet , R. Weissleder , Nat. Biomed. Eng. 2018, 2, 578. 31015631 10.1038/s41551-018-0236-8 PMC6192054

[13] N. J. Shah , A. J. Najibi , T. Y. Shih , A. S. Mao , A. Sharda , D. T. Scadden , D. J. Mooney , Nat. Biomed. Eng. 2020, 4, 40. 31937942 10.1038/s41551-019-0503-3

[14] R. M. Zemek , E. De Jong , W. L. Chin , I. S. Schuster , V. S. Fear , T. H. Casey , C. Forbes , S. J. Dart , C. Leslie , A. Zaitouny , M. Small , L. Boon , A. R. R. Forrest , D. O. Muiri , M. A. Degli‐Esposti , M. J. Millward , A. K. Nowak , T. Lassmann , A. Bosco , R. A. Lake , W. J. Lesterhuis , Sci. Transl. Med. 2019, 11, eaav7816. 31316010 10.1126/scitranslmed.aav7816

[15] Q. Chen , C. Wang , X. Zhang , G. Chen , Q. Hu , H. Li , J. Wang , D. Wen , Y. Zhang , Y. Lu , G. Yang , C. Jiang , J. Wang , G. Dotti , Z. Gu , Nat. Nanotechnol. 2019, 14, 89. 30531990 10.1038/s41565-018-0319-4

[16] C. Turnquist , B. M. Ryan , I. Horikawa , B. T. Harris , C. C. Harris , Cancer Cell 2020, 38, 598. 33038939 10.1016/j.ccell.2020.09.019 PMC7531591

[17] S. Y. Kim , S. Kim , J. E. Kim , S. N. Lee , I. W. Shin , H. S. Shin , S. M. Jin , Y. W. Noh , Y. J. Kang , Y. S. Kim , T. H. Kang , Y. M. Park , Y. T. Lim , ACS Nano 2019, 13, 12671. 31589013 10.1021/acsnano.9b04207

[18] M. Singh , H. Khong , Z. Dai , X. F. Huang , J. A. Wargo , Z. A. Cooper , J. P. Vasilakos , P. Hwu , W. W. Overwijk , J. Immunol. 2014, 193, 4722. 25252955 10.4049/jimmunol.1401160 PMC4201984

[19] D. Killock , Nat. Rev. Clin. Oncol. 2015, 12, 563. 10.1038/nrclinonc.2015.142 26305037

[20] M. S. Block , W. K. Nevala , Y. P. Pang , J. B. Allred , C. Strand , S. N. Markovic , Melanoma. Res. 2019, 29, 420. 30520800 10.1097/CMR.0000000000000556

[21] S. P. Kasturi , M. A. U. Rasheed , C. Havenar‐Daughton , M. Pham , T. Legere , Z. J. Sher , Y. Kovalenkov , S. Gumber , J. Y. Huang , R. Gottardo , W. Fulp , A. Sato , S. Sawant , S. Stanfield‐Oakley , N. Yates , C. LaBranche , S. M. Alam , G. Tomaras , G. Ferrari , D. Montefiori , J. Wrammert , F. Villinger , M. Tomai , J. Vasilakos , C. B. Fox , S. G. Reed , B. F. Haynes , S. Crotty , R. Ahmed , B. Pulendran , Sci. Immunol. 2020, 5. 10.1126/sciimmunol.abb1025 PMC8109745 32561559

[22] N. K. Routhu , N. Cheedarla , V. S. Bollimpelli , S. Gangadhara , V. V. Edara , L. Lai , A. Sahoo , A. Shiferaw , T. M. Styles , K. Floyd , S. Fischinger , C. Atyeo , S. A. Shin , S. Gumber , S. Kirejczyk , K. H. Dinnon 3rd , P. Y. Shi , V. D. Menachery , M. Tomai , C. B. Fox , G. Alter , T. H. Vanderford , L. Gralinski , M. S. Suthar , R. R. Amara , Nat. Commun. 2021, 12, 3587. 34117252 10.1038/s41467-021-23942-y PMC8196016

[23] E. Meylan , J. Tschopp , Mol. Cell 2006, 22, 561. 16762830 10.1016/j.molcel.2006.05.012

[24] M. V. Dhodapkar , M. Sznol , B. Zhao , D. Wang , R. D. Carvajal , M. L. Keohan , E. Chuang , R. E. Sanborn , J. Lutzky , J. Powderly , H. Kluger , S. Tejwani , J. Green , V. Ramakrishna , A. Crocker , L. Vitale , M. Yellin , T. Davis , T. Keler , Sci. Transl. Med. 2014, 6, 232ra51. 10.1126/scitranslmed.3008068 PMC6151129 24739759

[25] M. Hollmen , F. Roudnicky , S. Karaman , M. Detmar , Sci. Rep. 2015, 5, 9188. 25776849 10.1038/srep09188 PMC4361875

[26] X. Wang , Y. Lin , Acta Pharmacol. Sin. 2008, 29, 1275. 18954521 10.1111/j.1745-7254.2008.00889.x PMC2631033

[27] F. Allen , I. D. Bobanga , P. Rauhe , D. Barkauskas , N. Teich , C. Tong , J. Myers , A. Y. Huang , Oncoimmunology 2017, 7, e1393598. 29399390 10.1080/2162402X.2017.1393598 PMC5790335

[28] D. Wen , J. Wang , G. V. D. Driessche , Q. Chen , Y. Zhang , G. Chen , H. Li , J. Soto , M. Liu , M. Ohashi , Z. Wang , P. Abdou , Q. Hu , G. Dotti , S. Li , D. Fourches , Z. Gu , Matter 2019, 1, 1203.

[29] L. M. Arendt , J. McCready , P. J. Keller , D. D. Baker , S. P. Naber , V. Seewaldt , C. Kuperwasser , Cancer Res. 2013, 73, 6080. 23959857 10.1158/0008-5472.CAN-13-0926 PMC3824388

[30] A. M. Santander , O. Lopez‐Ocejo , O. Casas , T. Agostini , L. Sanchez , E. Lamas‐Basulto , R. Carrio , M. P. Cleary , R. R. Gonzalez‐Perez , M. Torroella‐Kouri , Cancers 2015, 7, 143. 25599228 10.3390/cancers7010143 PMC4381255

[31] T. Liang , D. Wen , G. Chen , A. Chan , Z. Chen , H. Li , Z. Wang , X. Han , L. Jiang , J. J. Zhu , Z. Gu , Adv. Mater. 2021, 33, 2100629. 10.1002/adma.202100629 33987883

[32] D. Hanahan , R. A. Weinberg , Cell 2011, 144, 646. 21376230 10.1016/j.cell.2011.02.013

[33] J. J. Havel , D. Chowell , T. A. Chan , Nat. Rev. Cancer 2019, 19, 133. 30755690 10.1038/s41568-019-0116-x PMC6705396

[34] B. Lim , W. A. Woodward , X. Wang , J. M. Reuben , N. T. Ueno , Nat. Rev. Cancer 2018, 18, 485. 29703913 10.1038/s41568-018-0010-y

[35] H. Hackstein , A. Knoche , A. Nockher , J. Poeling , T. Kubin , M. Jurk , J. Vollmer , G. Bein , Cell. Immunol. 2011, 271, 401. 21889130 10.1016/j.cellimm.2011.08.008

[36] H. Chi , C. Li , F. S. Zhao , L. Zhang , T. B. Ng , G. Jin , O. Sha , Front. Pharmacol. 2017, 8, 304. 28620298 10.3389/fphar.2017.00304 PMC5450331

[37] B. Desnues , A. B. Macedo , A. Roussel‐Queval , J. Bonnardel , S. Henri , O. Demaria , L. Alexopoulou , Proc. Natl. Acad. Sci. USA 2014, 111, 1497. 24474776 10.1073/pnas.1314121111 PMC3910605

[38] S. Aras , M. R. Zaidi , Br. J. Cancer 2017, 117, 1583. 29065107 10.1038/bjc.2017.356 PMC5729447

[39] E. Fuentes‐Mattei , G. Velazquez‐Torres , L. Phan , F. Zhang , P. C. Chou , J. H. Shin , H. H. Choi , J. S. Chen , R. Zhao , J. Chen , C. Gully , C. Carlock , Y. Qi , Y. Zhang , Y. Wu , F. J. Esteva , Y. Luo , W. L. McKeehan , J. Ensor , G. N. Hortobagyi , L. Pusztai , W. Fraser Symmans , M. H. Lee , S. C. Yeung , J. Natl. Cancer Inst. 2014, 106, 554. 10.1093/jnci/dju158 PMC4110474 24957076

[40] T. Suganami , J. Nishida , Y. Ogawa , Arterioscler. Thromb. Vasc. Biol. 2005, 25, 2062. 16123319 10.1161/01.ATV.0000183883.72263.13

[41] F. Hu , D. Huang , Y. Luo , P. Zhou , C. Lv , K. Wang , Q. Weng , X. Liu , Y. Guan , Y. Geng , J. Du , J. Chen , J. Wang , H. Wu , J. Immunother. Cancer e000498, 2020, 8. 32669292 10.1136/jitc-2019-000498 PMC7368548

[42] G. Chen , Z. Chen , D. Wen , Z. Wang , H. Li , Y. Zeng , G. Dotti , R. E. Wirz , Z. Gu , Proc. Natl. Acad. Sci. USA 2020, 117, 3687. 32029590 10.1073/pnas.1917891117 PMC7035610

