# Supplementary material for: Adipocytes Encapsulating Telratolimod Recruit and Polarize Tumor‐Associated Macrophages for Cancer Immunotherapy
Source: Adv Sci (Weinh). 2022 Dec 16;10(5):2206001. doi: 10.1002/advs.202206001 (PMC9929126; doi:10.1002/advs.202206001)
Supplement: Supplementary file 1 — Supporting Information [file ADVS-10-2206001-s001.pdf]

ADVANCED SCIENCE
Open Access

Supporting Information

for *Adv. Sci.*, DOI 10.1002/advs.202206001

Adipocytes Encapsulating Telratolimod Recruit and Polarize Tumor-Associated Macrophages for Cancer Immunotherapy

*Di Wen, Tingxizi Liang, Guojun Chen, Hongjun Li, Zejun Wang, Jinqiang Wang, Ruxing Fu, Xiao Han, Tianyuan Ci, Yuqi Zhang, Peter Abdou, Ruoxin Li, Linlin Bu, Gianpietro Dotti and Zhen Gu**

Supporting Information for

Adipocytes Encapsulating Telratolimod Recruit and Polarize Tumor-associated Macrophages for Cancer Immunotherapy

Di Wen,[1, 2] *Tingxizi Liang,*[1,4] *Guojun Chen,*[1] *Hongjun Li,*[1,4] *Zejun Wang,*[1] *Jinqiang Wang,* [1,4] *Ruxing Fu,*[1] *Xiao Han,*[1] *Tianyuan Ci,*[1] *Yuqi Zhang,*[1,4] *Peter Abdou,*[1] *Ruoxin Li,*[1] *Linlin Bu,*[1] *Gianpietro Dotti,*[3] *Zhen Gu*[1, 4, 5, 6*]

1. Department of Bioengineering, University of California, Los Angeles, California 90095, USA
2. Earle A. Chiles Research Institute, Robert W. Franz Cancer Center, Providence Portland Medical Center, Portland, Oregon, 97213, USA
3. Department of Microbiology and Immunology, School of Medicine, University of North Carolina at Chapel Hill, Chapel Hill, NC 27599, USA
4. Key Laboratory of Advanced Drug Delivery Systems of Zhejiang Province, College of Pharmaceutical Sciences, Zhejiang University, Hangzhou, 310058, China
5. Jinhua Institute of Zhejiang University, Jinhua 321299, China
6. Department of General Surgery, Sir Run Run Shaw Hospital, School of Medicine, Zhejiang University, Hangzhou 310016, China

[*]Correspondence: guzhen@zju.edu.cn (Z.G.)

This Supporting Information includes:

Figure S1. B16F10 and telratolimod promote macrophage polarization and inflammatory cytokine secretion.

Figure S2. Resiquimod and Telratolimod have similar effects on macrophage polarization.

Figure S3. Phagocytosis of B16F10 by macrophage was monitored by confocal microscope.

Figure S4. Cytotoxicity of telratolimod and characterization of telratolimod@adipocytes.

Figure S5. Adipokine array of telratolimod@adipocyte cocultured with B16F10.

Figure S6. Effect of FABP4 inhibitor on telratolimod@adipocyte induced macrophage polarization.

Figure S7. Effect of telratolimod on macrophage polarization during the crosstalk between cancer cell and immune cell.

Figure S8. Effect of telratolimod on Treg population in the presence of cancer cells and immune cells.

Figure S9. Pharmacokinetics profile of telratolimod after intratumoral injection.

Figure S10. Representative immunofluorescent staining of immune cells in tumor.

Figure S11. Serum cytokine level in primary tumor model after administration of adipocyte and telratolimod.

Figure S12. Primary tumor growth and immune activity in B16F10 melanoma distant tumor model.

Figure S13. Representative H&E staining of each organ after treatment.

Figure S14. Maturation of dendritic cells in tumor-draining lymph node.

Figure S15. Serum cytokine level in distant tumor model after administration of adipocyte and telratolimod.

Figure S16. Primary tumor growth and immune activity in 4T1 mammary carcinoma distant tumor model.

Figure S17. Distant tumor growth and immune activity in 4T1 mammary carcinoma distant tumor model.

Figure S18. Complete blood count in distant 4T1 mammary carcinoma model.

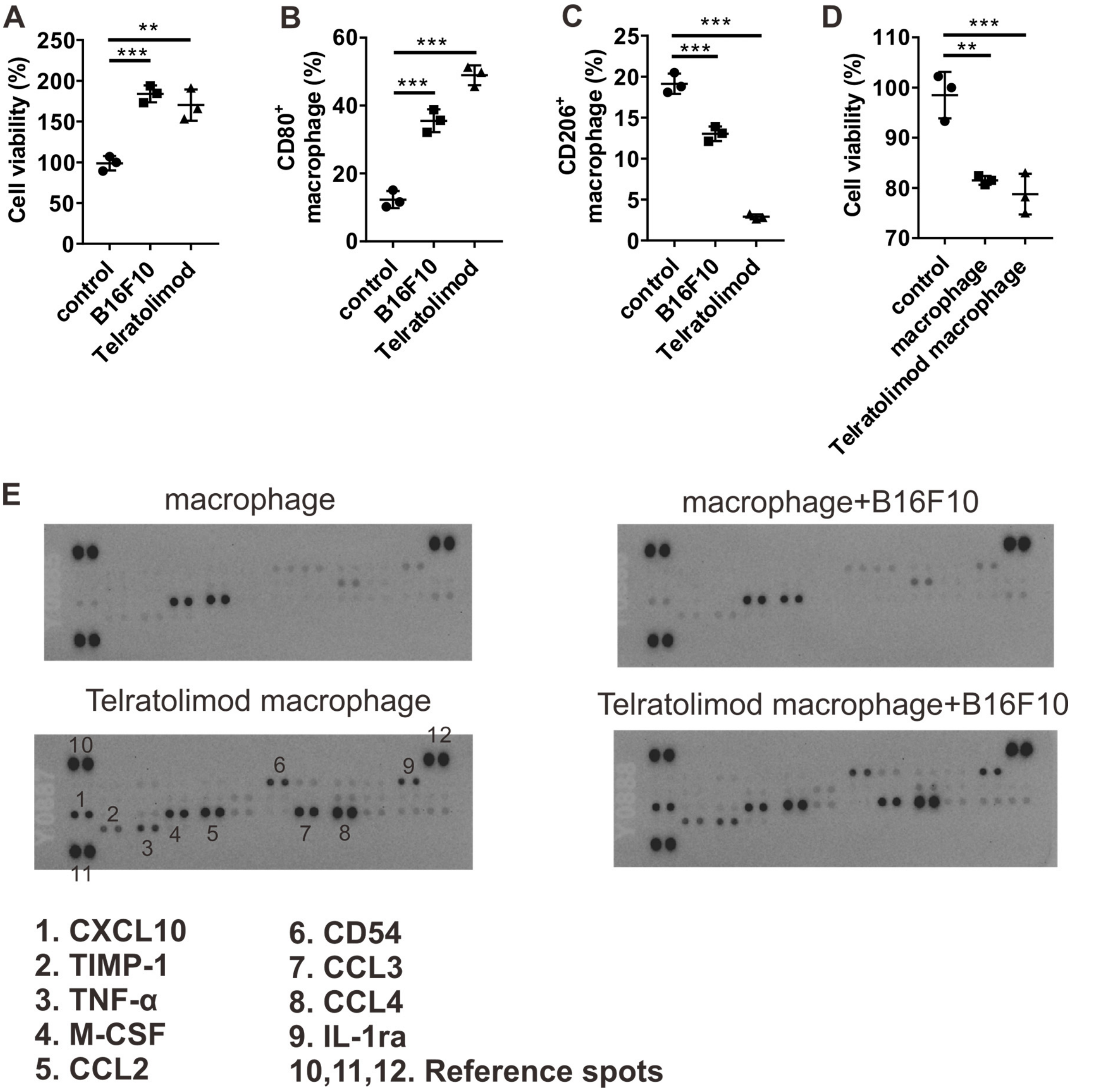

Figure S1. B16F10 and telratolimod promote macrophage polarization and secretion of inflammatory cytokine. (**A**) B16F10 and telratolimod promote macrophage proliferation. (**B, C**) B16F10 and telratolimod promote the M1 polarization of macrophage (**B**) while decrease the M2 polarization (**C**). (**D**) macrophage or telratolimod treated macrophage suppress B16F10 growth in a transwell system. Bars are presented as means ± SD (n = 3). One-way ANOVA with a Tukey post-hoc test was performed. **$p < 0.01$, ***$p < 0.001$. (**E**) The inflammatory cytokine secretion was determined by cytokine array study.

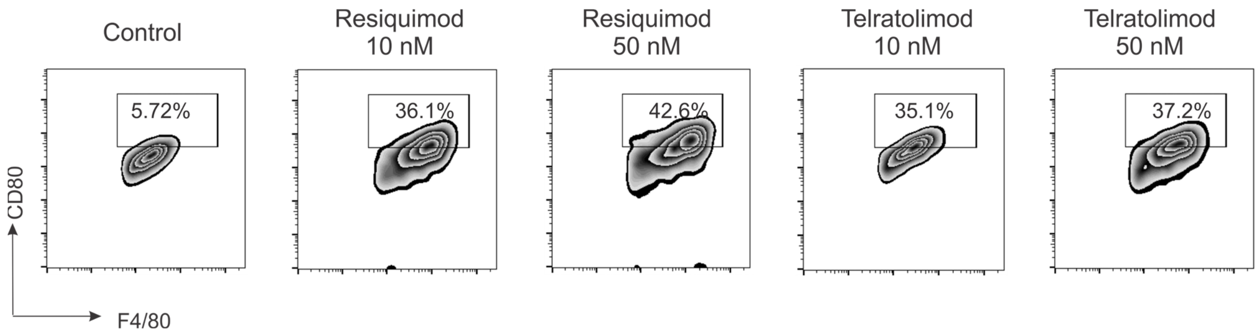

Figure S2. Resiquimod and Telratolimod have similar effects on macrophage polarization.

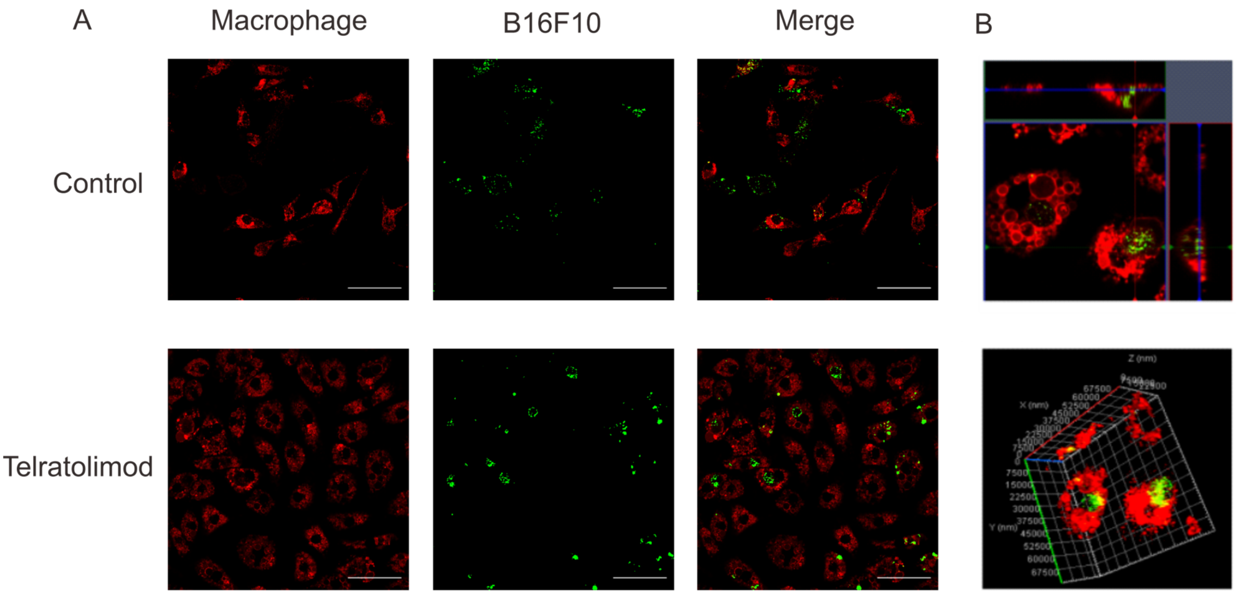

Figure S3. Phagocytosis of B16F10 by macrophage was monitored by confocal microscope in 2D (**A**) and 3D (**B**) scope. Scale bar: 20 μm.

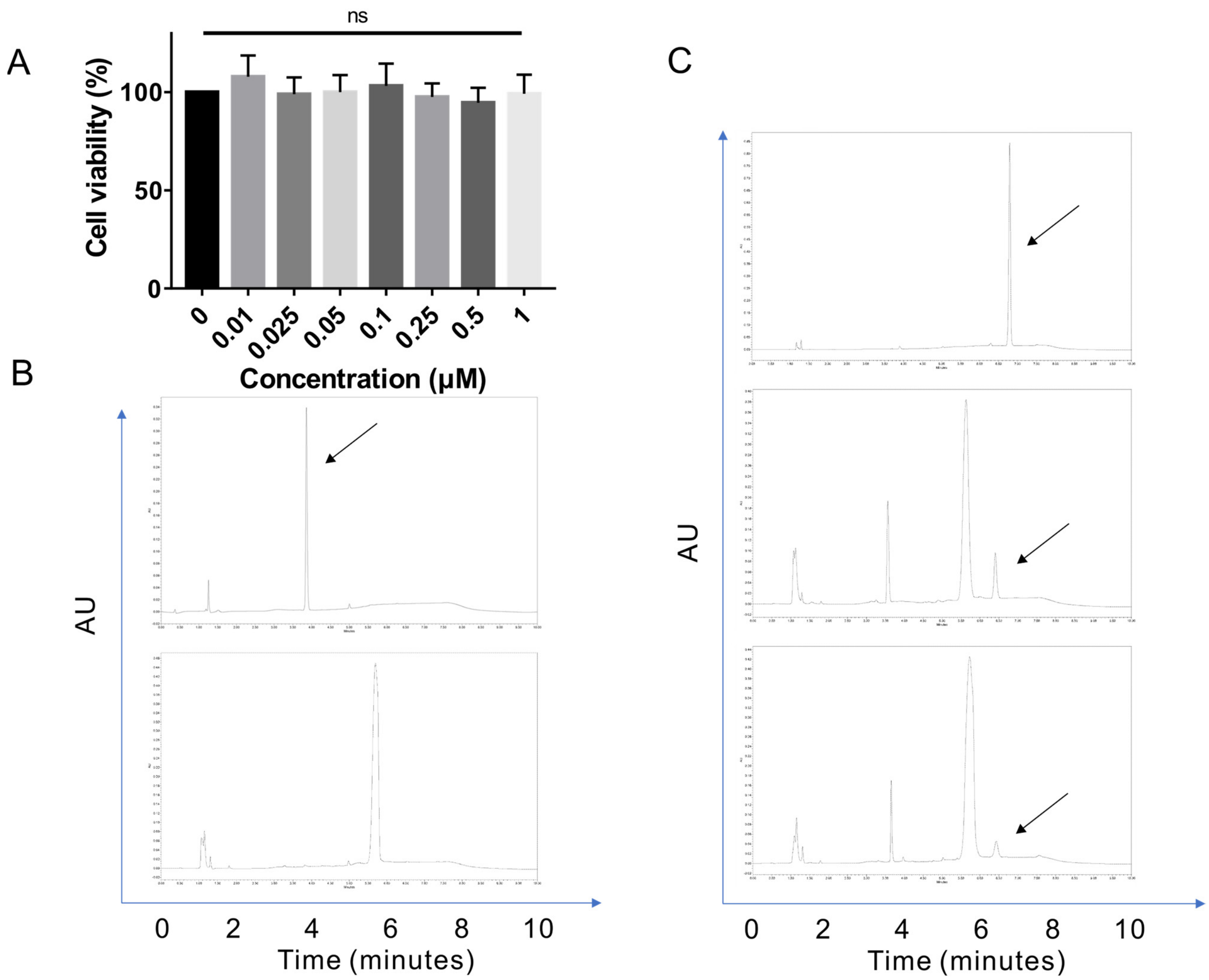

Figure S4. Cytotoxicity of telratolimod and characterization of telratolimod@adipocytes. (**A**) Cytotoxicity of telratolimod toward B16F10. Error bars are presented as means ± SD (n = 3). (**B**) Loading capacity of resiquimod in adipocytes. Upper: standard HPLC spectrum of resiquimod. Lower: HPLC spectrum of adipocyte after resiquimod encapsulation. (**C**) Loading and stability of telratolimod in adipocytes. Upper: standard HPLC spectrum of telratolimod. Middle: HPLC spectrum of adipocyte after telratolimod encapsulation at day 0. Lower: HPLC spectrum of adipocyte after telratolimod encapsulation at day 1.

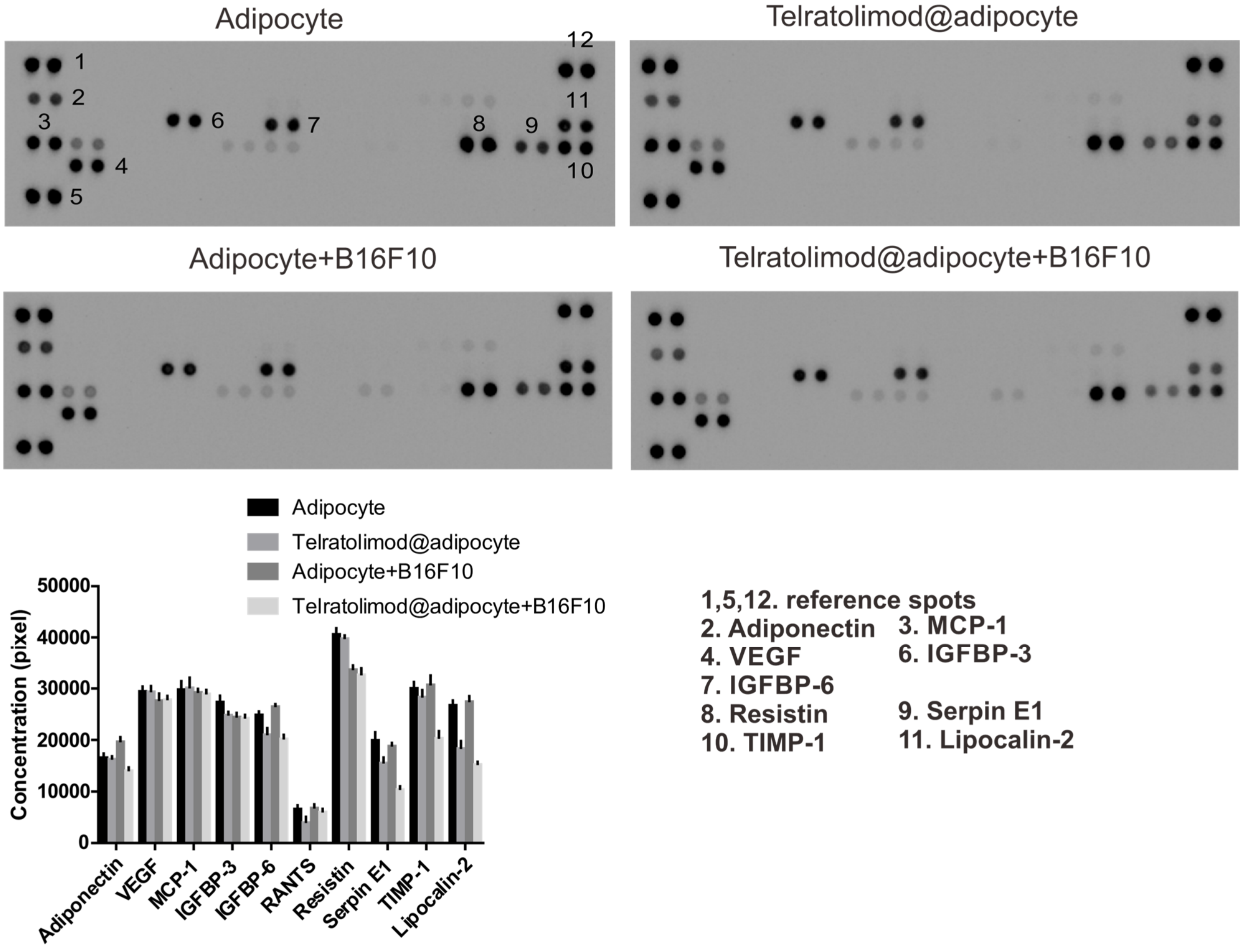

Figure S5. Adipokine array of telratolimod@adipocyte cocultured with B16F10. Error bars are presented as means ± SD ($n = 3$).

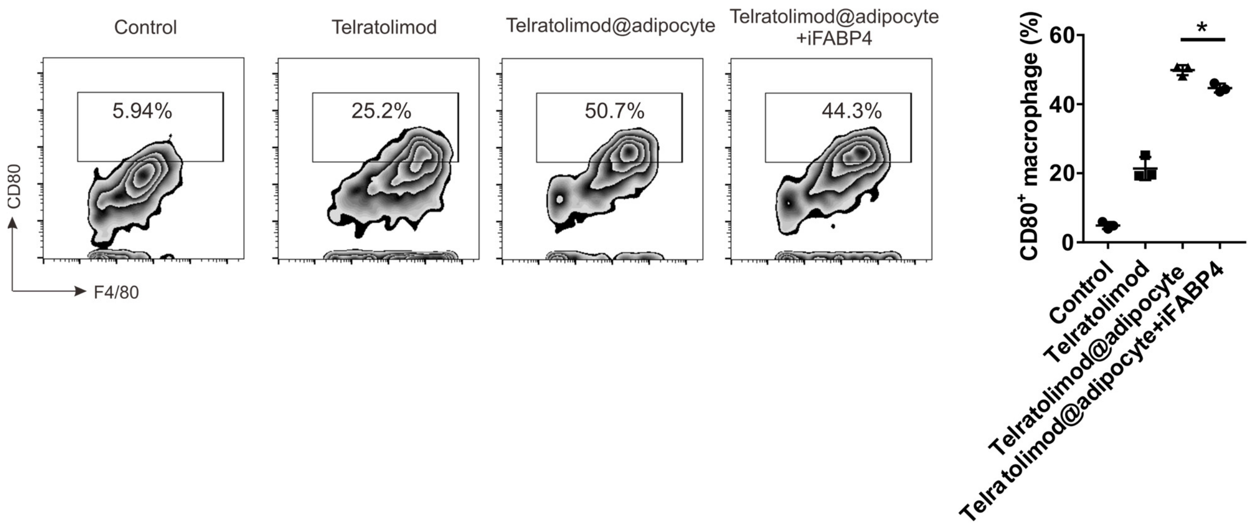

Figure S6. Effect of FABP4 inhibitor on telratolimod@adipocyte induced macrophage polarization. Bars are presented as means ± SD (n = 3). One-way ANOVA with a Tukey post-hoc test was used for the analysis. *$p < 0.05$.

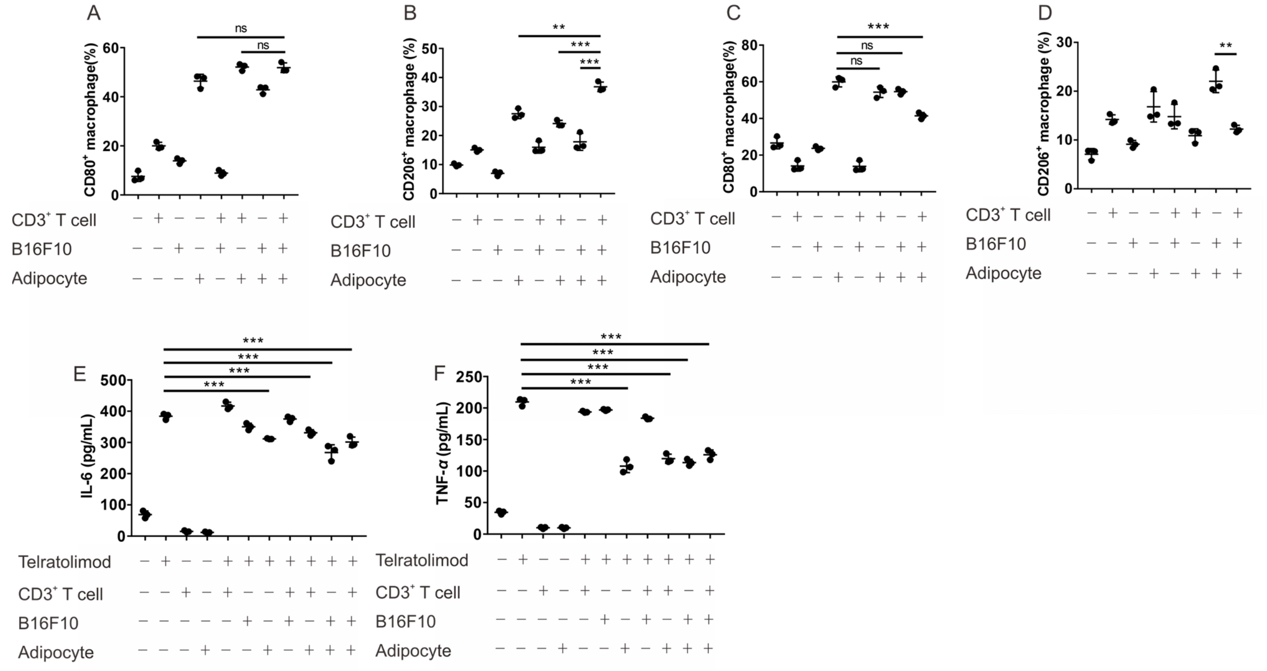

Figure S7. Effect of telratolimod on macrophage polarization in the presence of cancer cells and immune cells. (**A**, **B**) Effect of different cells on the M1 (**A**) and M2 (**B**) polarization of macrophages. (**C, D**) Effect of telratolimod on M1 (**C**) and M2 (**D**) polarization of macrophages in the presence of cancer cells and immune cells. (**E, F**) Effect of telratolimod on cytokine secretion in the presence of cancer cells and immune cells. Bars are presented as means ± SD (n = 3). One-way ANOVA with a Tukey post-hoc test was performed. $^{**}p < 0.01$, $^{***}p < 0.001$.

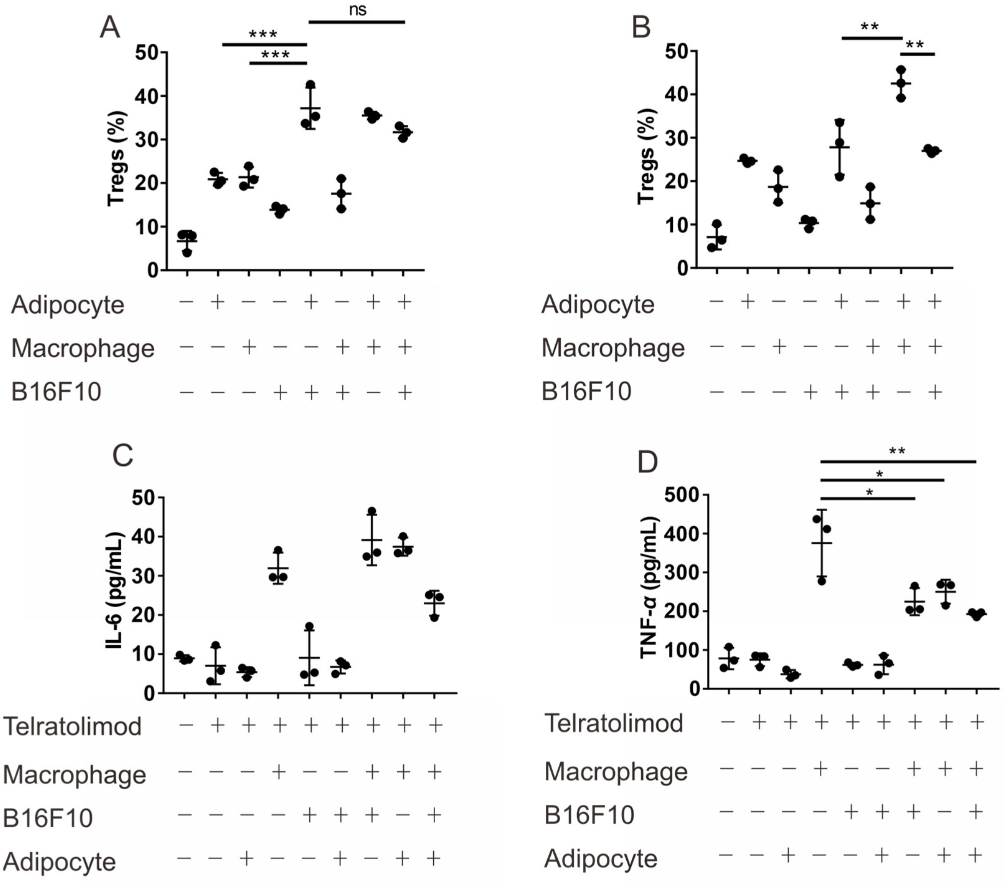

Figure S8. Effect of telratolimod on Treg population in the presence of cancer cells and immune cells. (**A**) Effect of different cells on Tregs. (**B**) Effect of telratolimod on Tregs in the presence of cancer cells and immune cells. (**C, D**) Effect of telratolimod on cytokine secretion in the presence of cancer cells and immune cells. Bars are presented as means ± SD ($n = 3$). One-way ANOVA with a Tukey post-hoc test was performed. $^*p < 0.05$, $^{**}p < 0.01$, $^{***}p < 0.001$.

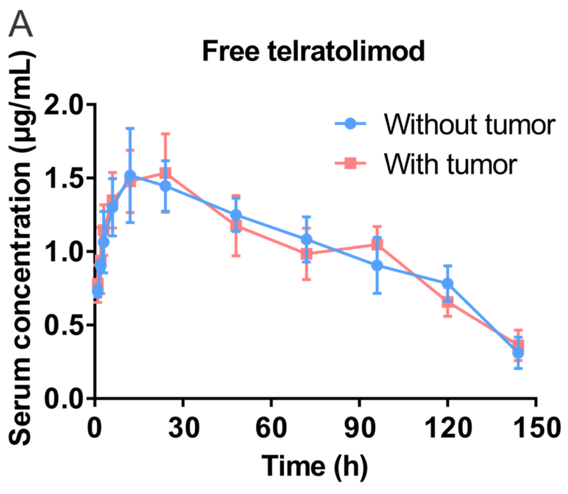

Figure S9. Pharmacokinetics profile of telratolimod after intratumoral injection. (**A**) Free drug. (**B**) Telratolimod encapsulated in adipocytes. Bars are presented as means ± SD (n = 3).

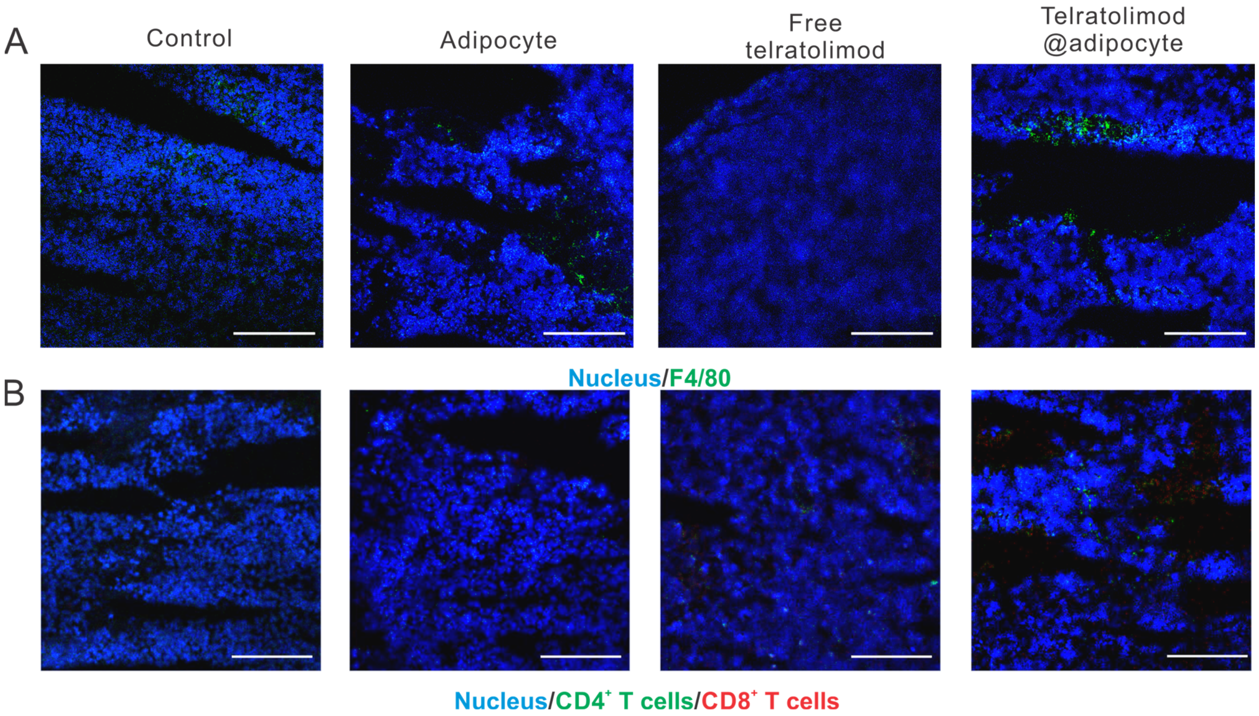

Figure S10. Representative immunofluorescence staining of immune cells within tumor. (**A**) macrophages. (**B**) $CD4^+$ and $CD8^+$ T cells. Scale bar: 200 μm.

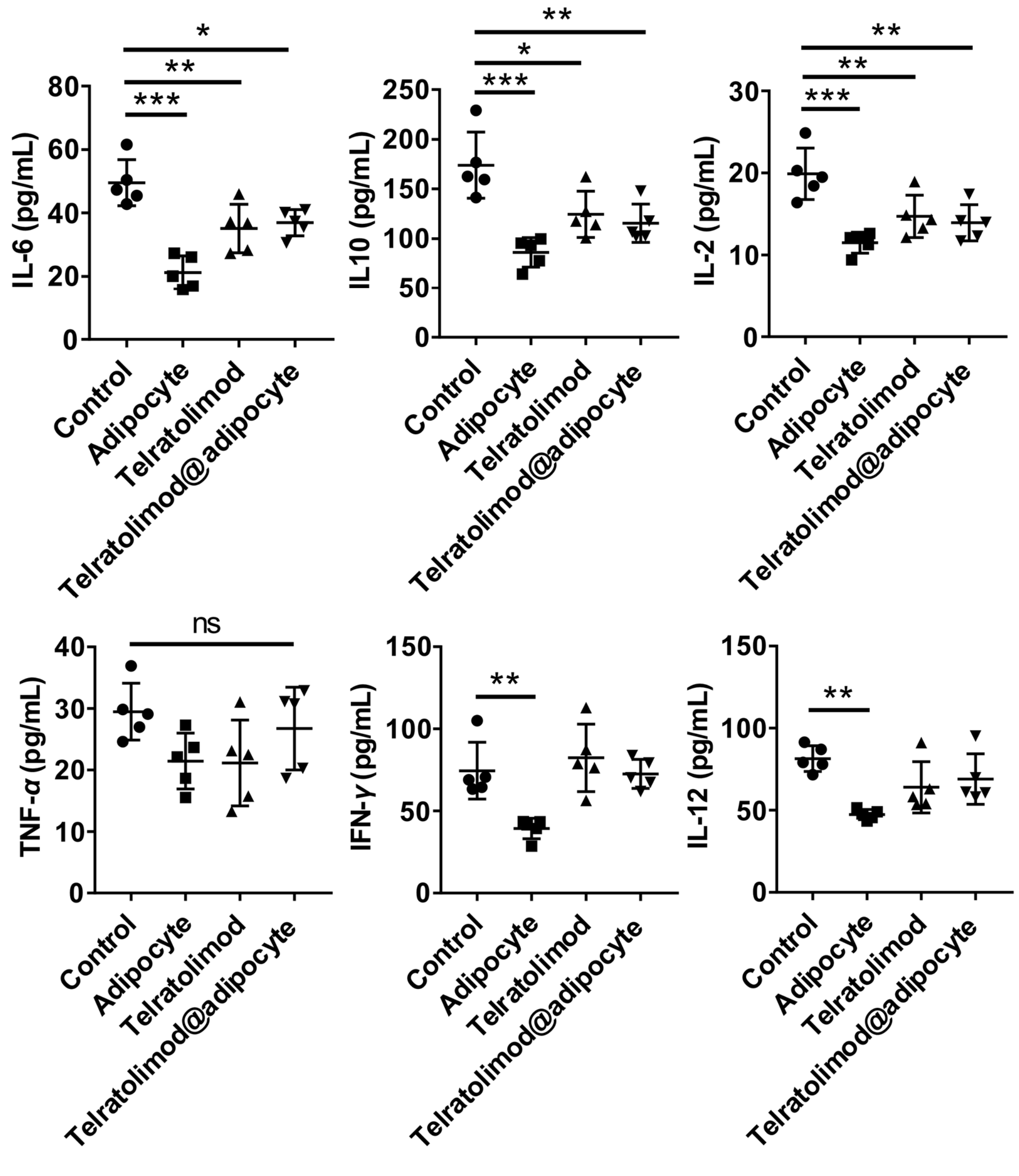

Figure S11. Serum cytokine level in primary tumor model after administration of adipocytes and telratolimod (n = 5). One-way ANOVA with a Tukey post-hoc test was used for the analysis. *p < 0.05, **p < 0.01, ***p < 0.001.

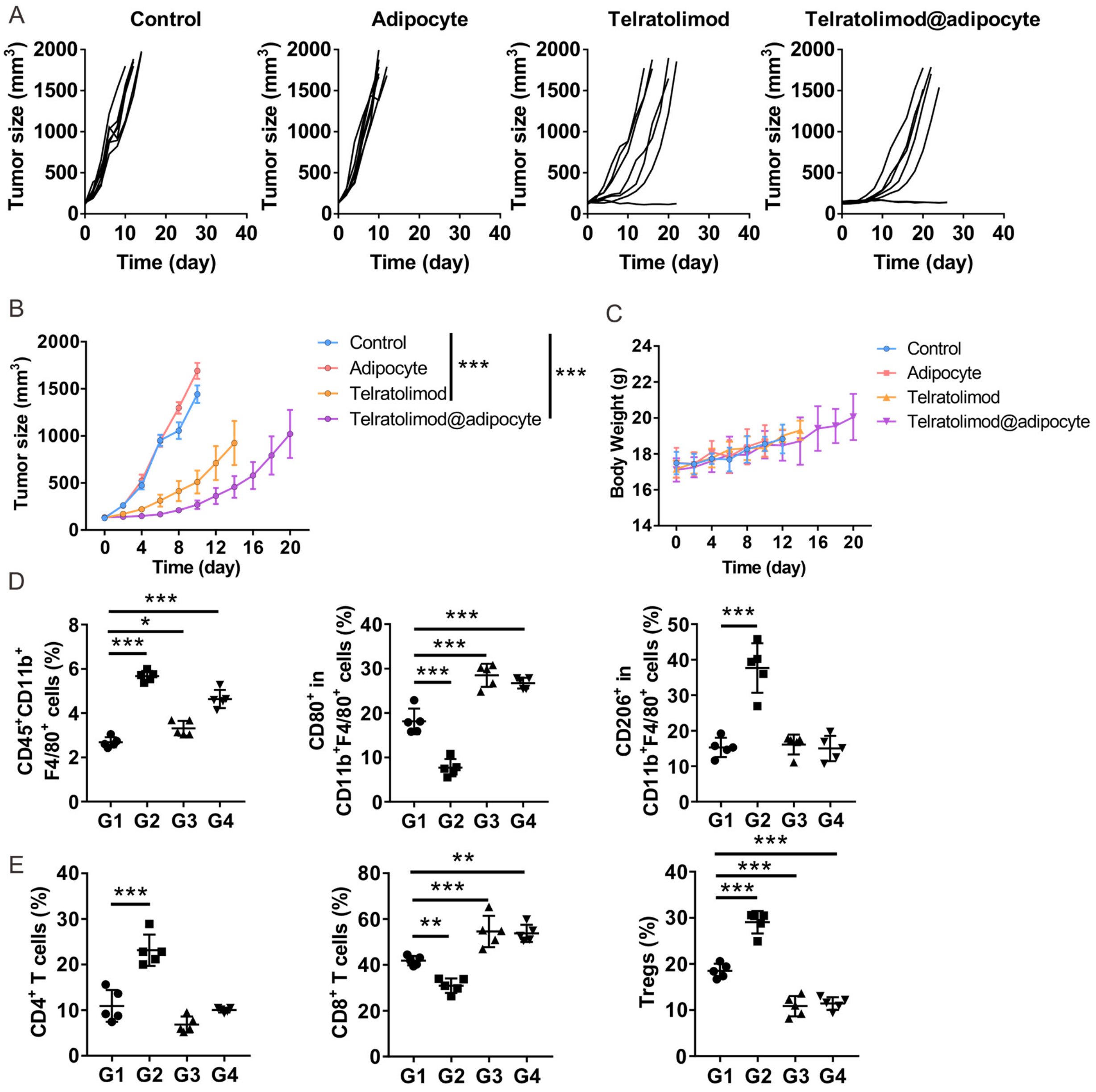

Figure S12. Primary tumor growth and immune activity in B16F10 melanoma distant tumor model. (**A**) Individual primary tumor growth. (**B**) Average primary tumor size in each group. Data are presented as means ± SEM ($n = 7$). (**C**) Average body weight of the mice in each experimental group. (**D**) Quantification of macrophages in primary tumors. (**E**) Quantification of T cells in primary tumors. G1: Control. G2: Adipocytes. G3: Telratolimod (0.2 mg/kg). G4: Telratolimod@adipocytes. Data are presented as means ± SD ($n = 5$). Statistical significance was calculated *via* one-way ANOVA analysis with a Tukey post-hoc test. $^{*}p < 0.05$, $^{**}p < 0.01$, $^{***}p < 0.001$.

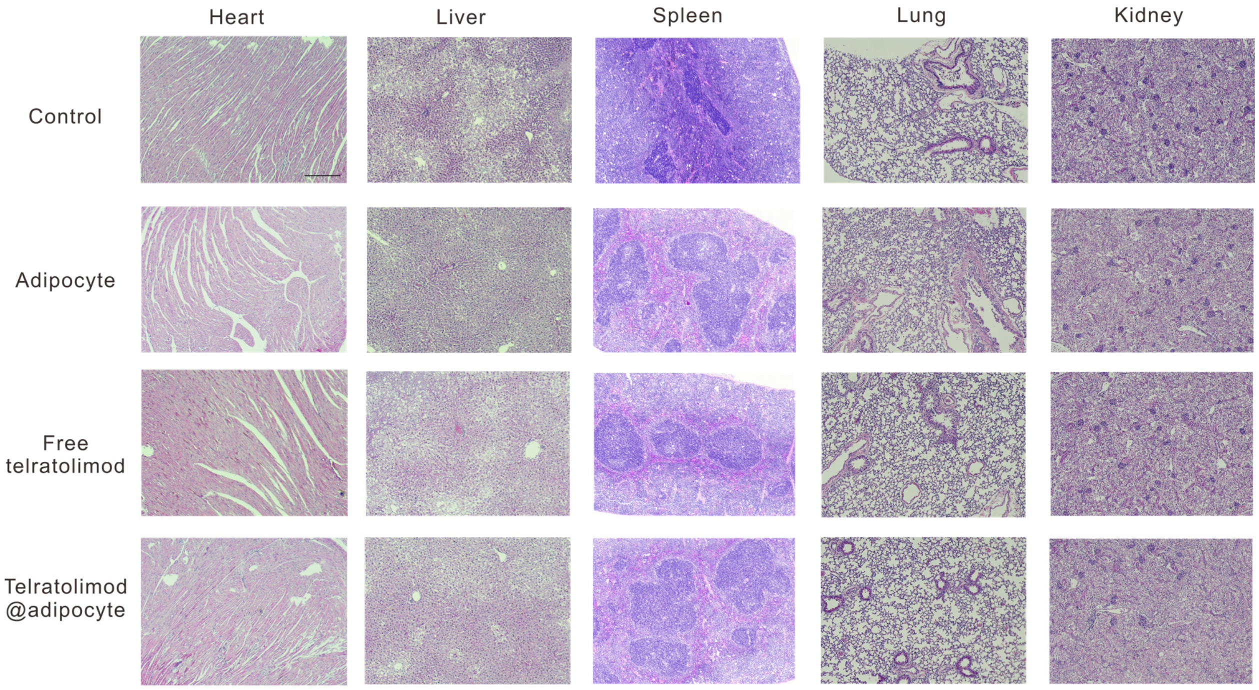

Figure S13. Representative H&E staining of each organ after treatment. Scale bar: 200 μm.

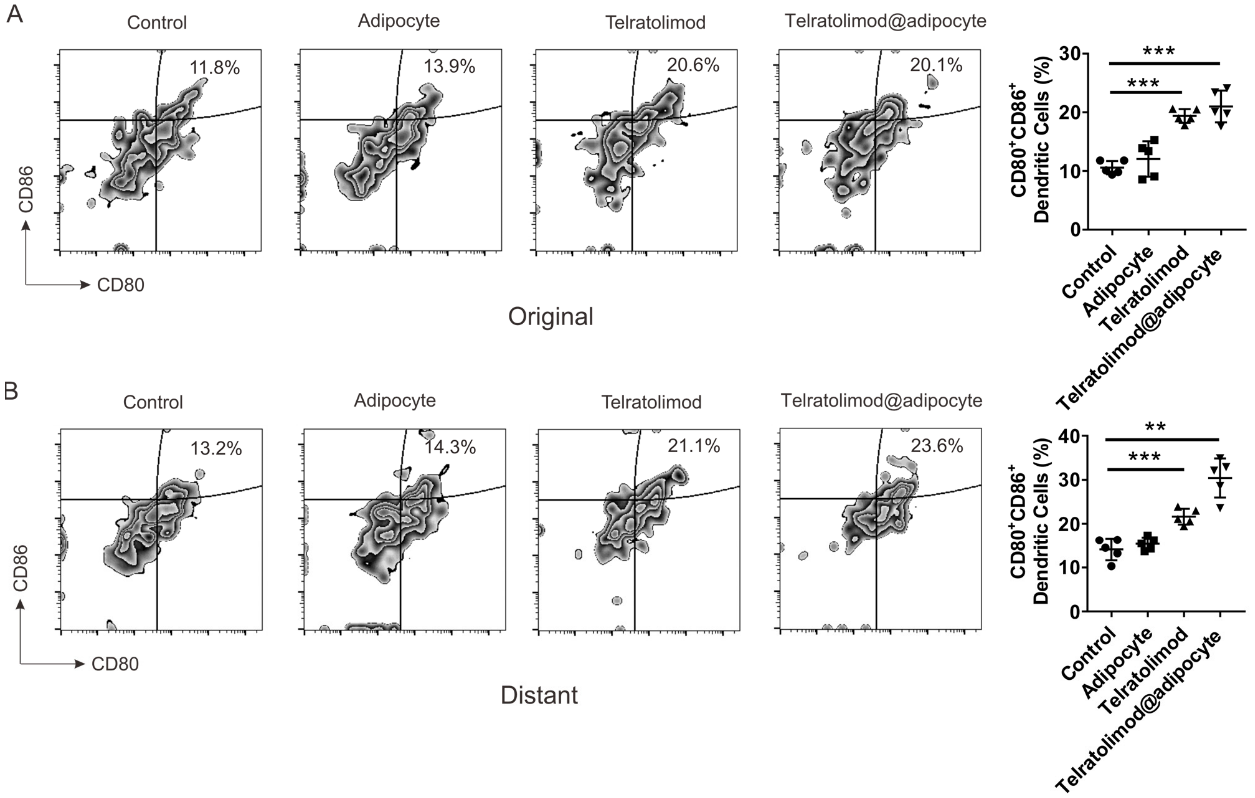

Figure S14. Maturation of DCs in the tumor-draining lymph node. (**A**) Primary tumors. (**B**) Distant tumors. $n = 5$. One-way ANOVA with a Tukey post-hoc test was used for the analysis. $^{**}p < 0.01$, $^{***}p < 0.001$.

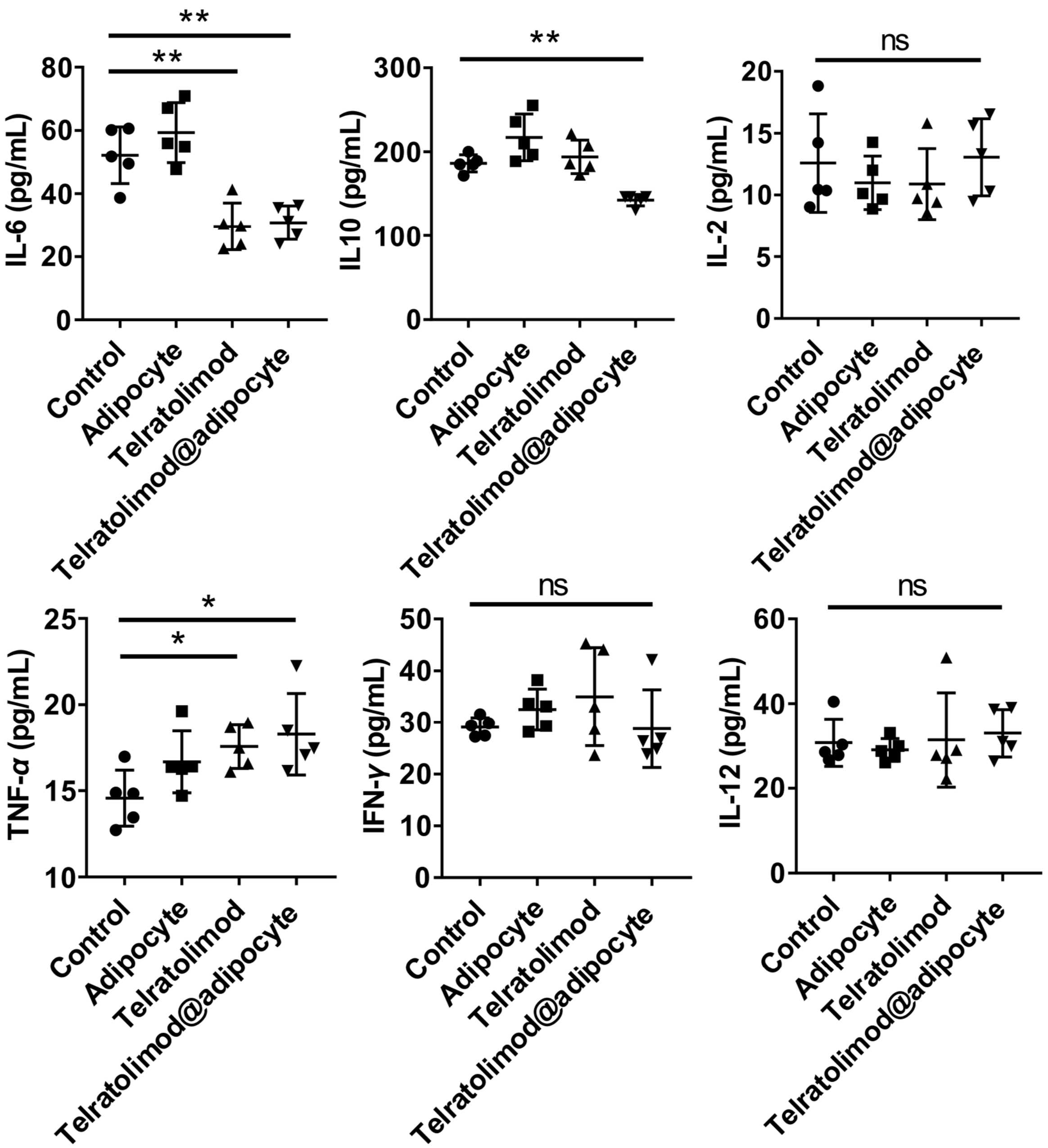

Figure S15. Serum cytokine level in distant tumor model after the administration of adipocytes and telratolimod ($n = 5$). One-way ANOVA with a Tukey post-hoc test was used for the analysis. *$p < 0.05$, **$p < 0.01$.

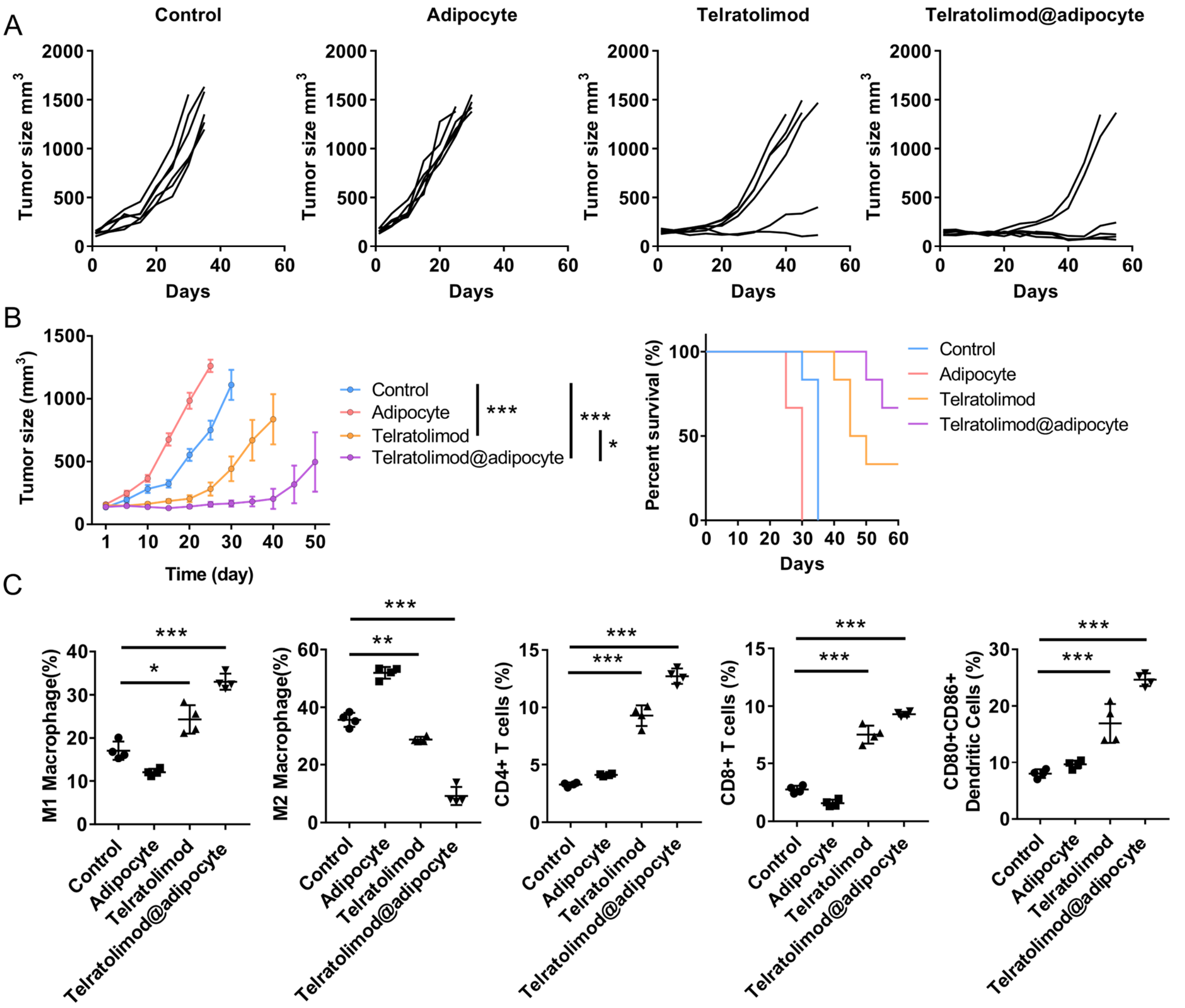

Figure S16. Primary tumor growth and immune activity in 4T1 mammary carcinoma distant tumor model. (**A**) Individual primary tumor growth. (**B**) Average primary tumor size and mouse survival in each group. Data are presented as means ± SEM (n = 6). (**C**) Quantification of macrophages, T cells, and dendritic cells in primary tumors. Data are presented as means ± SD (n = 4). Statistical significance was calculated *via* one-way ANOVA analysis with a Tukey post-hoc test. *$p < 0.05$, **$p < 0.01$, ***$p < 0.001$.

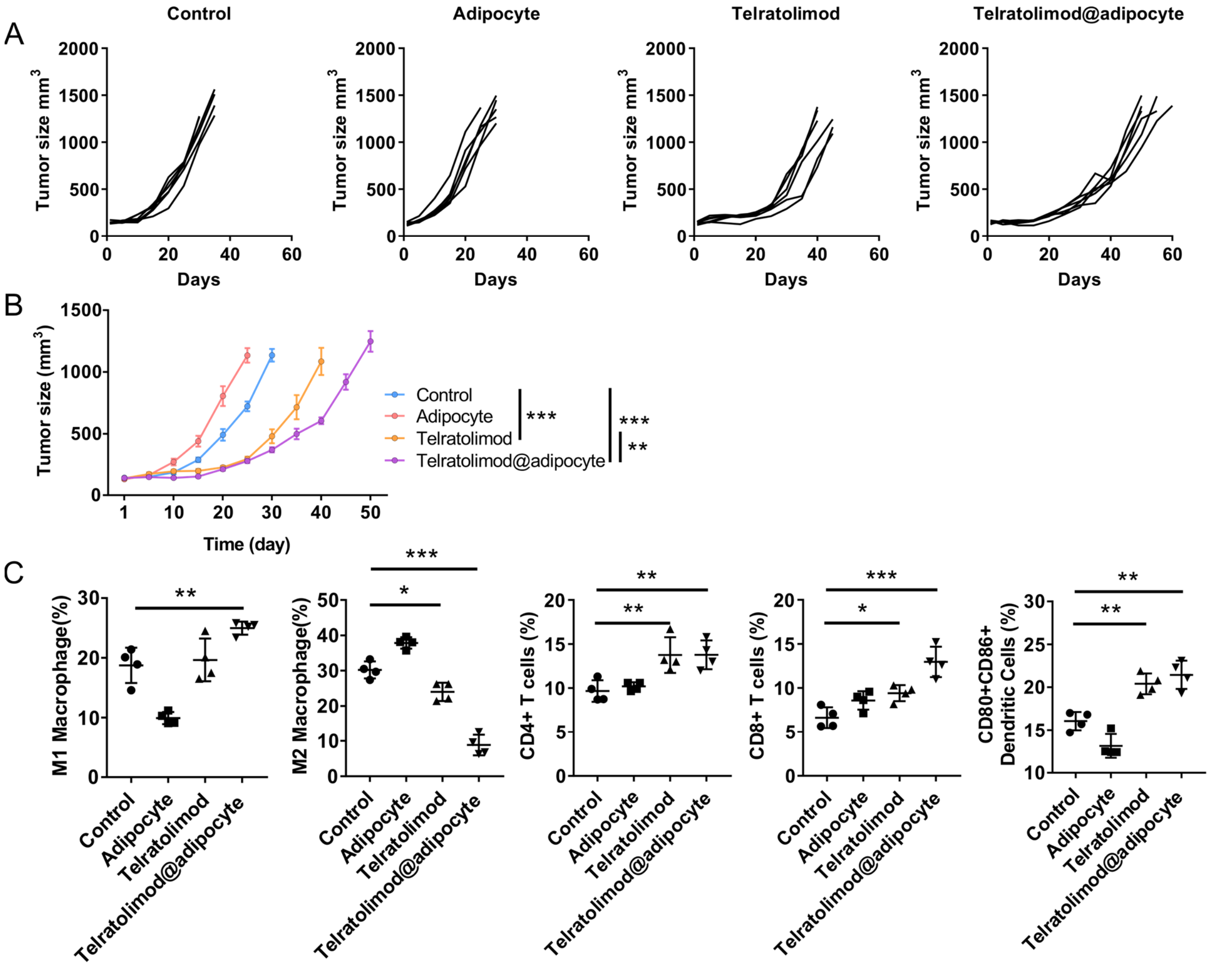

Figure S17. Distant tumor growth and immune activity in 4T1 mammary carcinoma distant tumor model. (**A**) Individual primary tumor growth. (**B**) Average primary tumor size and mouse survival in each group. Data are presented as means ± SEM (n = 6). (**C**) Quantification of macrophages, T cells, and dendritic cells in distant tumors. Data are presented as means ± SD (n = 4). Statistical significance was calculated *via* one-way ANOVA analysis with a Tukey post-hoc test. *$p < 0.05$, **$p < 0.01$, ***$p < 0.001$.

	Control	Adipocyte	Free MEDI9197	Adipocyte MEDI9197
WBC (rf: 5.69-14.84 k/uL)	172.02±22.37	168.46±11.36	204.74±28.61	192.33±25.17
RBC (rf: 8.16-11.69 M/uL)	8.36±1.23	8.89±0.37	9.62±1.53	8.49±0.23
HGB (rf: 12.4-18.9 g/dL)	13.3±1.8	14.6±0.9	12.7±0.8	13.0±1.4
HCT (rf: 43.5-67%)	42.77±2.57	43.63±3.33	43.90±1.50	46.30±0.80
MCV (rf: 50.8-64.1 fL)	54.8±1.2	55.1±1.4	55.3±1.7	55.2±0.4
MCH (rf: 13-17.6 pg)	15.1±0.3	14.5±0.4	14.8±0.7	14.6±0.2
MCHC (rf: 23.9-33.1 g/dL)	27.0±1.8	28.1±0.8	28.9±0.7	27.8±1.4
PLT (rf: 476-1611 K/uL)	736±162	613±67	645±84	707±133

Fig S18. Complete blood count in distant 4T1 mammary carcinoma model. Mice were sacrificed one week after the final treatment. Complete blood counts include white blood cells (WBC), red blood cells (RBC), hemoglobin (HGB), hematocrit (HCT), mean corpuscular volume (MCV), mean corpuscular hemoglobin (MCH), mean corpuscular hemoglobin concentration (MCHC) and platelet (PLT). Data are presented as mean ± SD. (*n*=3). Reference ranges of hematology data of healthy female Balb/c mice were obtained from Charles River Laboratories:

(https://www.criver.com/sites/default/files/resources/BALBcMouseClinicalPathologyData.pdf).
